# Incidence of mental disorders in the general population aged 1–30 years disaggregated by gender and socioeconomic status

**DOI:** 10.1007/s00127-023-02425-z

**Published:** 2023-01-24

**Authors:** Javier Mar, Igor Larrañaga, Oliver Ibarrondo, Ana González-Pinto, Carlota las Hayas, Ane Fullaondo, Irantzu Izco-Basurko, Jordi Alonso, Iñaki Zorrilla, Gemma Vilagut, Maider Mateo-Abad, Esteban de Manuel, the UPRIGHT Consortium, Nerea González, Patricia Pérez Martínez, Itziar Vergara, Jessica Fernández-Sevillano, Silvia Gabrielli, Silvia Rizzi, Antoni Zwiefka, Dominik Krzyżanowski, Iwona Mazur, Luba Jakubowska, Renata Poteralska, Piotr Czyż, Urszula Andruszko, Paweł Błasiak, Katarzyna Krajewska, Grzegorz Pytlarz, Ilona Szczygieł-Grüdl, Odin Hjemdal, Roxanna Morote, Frederick Anyan, Dora Gudrun Gudmundsdottir, Solveig Karlsdottir, Hans Henrik Knoop, Mette Marie Ledertoug, Louise Tidmand, Anna Sigridur Olafsdottir, Unnur B Arnfjord, Bryndis Jona Jonsdottir

**Affiliations:** 1 Osakidetza Basque Health Service, Research Unit, Debagoiena Integrated Health Organisation, Hospital ‘Alto Deba’, Unidad de Gestión Sanitaria, Avenida Navarra 16, 20500 Arrasate-Mondragón, Spain; 2 Biodonostia Health Research Institute, Donostia-San Sebastián, Spain; 3 REDISSEC (Health Services Research on Chronic Patients Network), Bilbao, Spain; 4 Kronikgune Institute for Health Services Research, Barakaldo, Spain; 5 Osakidetza Basque Health Service, Araba University Hospital, Vitoria-Gasteiz, Spain; 6 UPV/EHU-University of the Basque Country, Vitoria-Gasteiz, Spain; 7 CIBERSAM, CIBER en Salud Mental, Madrid, Spain; 8 Bioaraba Health Research Institute, Vitoria-Gasteiz, Spain; 9 Health Services Research Group, IMIM-Institut Hospital del Mar d’Investigacions Mèdiques, Barcelona, Spain; 10 CIBERESP, CIBER en Epidemiología y Salud Pública, Madrid, Spain; 11 UPF-Pompeu Fabra University, Barcelona, Spain

**Keywords:** Adolescent, Mental disorders, Healthcare disparities, Socioeconomic factors, Incidence, Adolescent psychiatry, Child psychiatry

## Abstract

**Purpose:**

The objective of this study was to estimate the incidence and age of onset of mental disorders diagnosed by gender and socioeconomic status (SES) in children, adolescents, and young adults up to 30 years of age in the whole population of the Basque Country (Spain).

**Methods:**

All mental health diagnoses documented in Basque Health Service records from 1 January 2003 to 31 December 2018, were classified into eight clusters: anxiety, attention deficit hyperactivity disorder (ADHD), conduct disorders, depression, psychosis/personality disorders, substance use, eating disorders, and self-harm. We calculated incidence and cumulative incidence for each cluster, disaggregated by gender, and socioeconomic status (SES). Poisson regression analyses were performed.

**Results:**

Overall, 9,486,853 person-years of observation were available for the 609,281 individuals included. ADHD and conduct disorders were diagnosed in the first decade, anxiety and depression disorders in the second and third decades, and psychosis/personality and substance use in the third. The cumulative incidence at 18 years of age for any type of disorder was 15.5%. The group with low SES had a statistically significantly higher incidence of all eight clusters. The incidence of ADHD, conduct disorders, depression, psychosis/personality disorders, and substance use was higher in males and that of anxiety, eating disorders and self-harm was higher in females.

**Conclusions:**

The incidence of mental disorders is high among children, adolescents, and young adults in the Basque Country underlining the need for preventive interventions. Marked differences by gender and SES highlight mental health inequalities, especially for depression and psychosis in low SES males.

**Supplementary Information:**

The online version contains supplementary material available at 10.1007/s00127-023-02425-z.

## Introduction

Knowledge about the age of onset of mental disorders is critical to preventing adverse conditions experienced during childhood and adolescence determining poor levels of mental health in adulthood [1–6]. The lifetime prevalence of some mental disorders varies by gender and/or socioeconomic level [7], and may reach as high as 50% in individuals between 20 and 30 years old [8]. Approximately half of all mental disorders begin in adolescence and three-quarters by the age of 25 [8, 9].

In recent years, numerous interventions have been developed for the prevention of mental disorders in adolescence [10–13]. Each country presents a specific epidemiological context to be taken into account in the planning and implementation of preventive mental health policies. Knowing the baseline epidemiology of mental disorders is the basis for evaluating the effectiveness of population programs.

Most mental health epidemiological studies are based on surveys that rely on self-reporting of the presence of psychiatric symptoms [14]. Self-reported symptoms are converted into codes from the successive versions of the Diagnostic and Statistical Manual of Mental Disorders and the International Classification of Diseases, to estimate prevalence indicators (past year prevalence or lifetime prevalence) [8, 15]. Surveys also collect information from people with problems who do not contact health services but may be subject to response call bias, a type of selection bias, and this raises concerns about the validity of the diagnosis [16, 17].

Historically, mental health registries have not been a feasible basis for such research due to the limited number of hospital admissions and the tradition of psychiatric care being managed separately from other public health services. In other fields like cancer, however, studies based on population registries have been key in the recognition of cancer as a priority public health problem and have promoted the development of preventive policies [18, 19]. Currently, the creation of databases containing the information in populations’ electronic medical records offers a new source of information for mental health research [16, 17]. Specifically, the diagnoses classified using the International Classification of Diseases, Ninth and Tenth Revision (ICD-9 or ICD-10) codes can be used as an indicator of the incidence of mental disorders. Based on Danish population records, it has been estimated that the cumulative incidence of mental disorders at 18 years of age is between 11.02% [16] and 15.01% [17].

The literature provides evidence of excess risks of mental disorders among more deprived populations [20, 21]. This, together with the well-known existence of large inequalities in the mental health of young people and adolescents, underlines the need for differentiated analysis of the epidemiology by gender and socioeconomic status (SES) [22]. Low SES children and adolescents reported two to three times more mental disorders, the correlation between different types of mental disorders varying with age and being heterogeneous with gender [22]. Nonetheless, the published analyses of data from registries have only disaggregated the risk by gender [16, 17]. Therefore, the stratification of incidence of mental disorders by both gender and SES would advance efforts to make its prevention a priority in public health.

The objective of this study was to estimate the incidence and age of onset of mental disorders diagnosed in healthcare settings by gender and SES in children, adolescents and young adults up to 30 years of age in the whole population of the Basque Country (Spain).

## Methods

This study is part of a larger research project called UPRIGHT [23, 24] funded by the European Union’s Horizon 2020 innovation and research programme (grant agreement number: 754919). The funding body had no role in the study design, writing of the protocol or the decision to submit the paper for publication. The protocol of the study was approved by the Clinical Research Ethics Committee of the Basque Country (number PI2019078).

We carried out a retrospective observational study to calculate the incidence and cumulative incidence of mental disorders by diagnostic group. For this, we used the population registry of the Basque Health Service’s institutional database, Oracle Business Intelligence (OBI), which contains anonymised administrative and clinical records from 1 January 2003 to 31 December 2018 [25]. Over that period, the total Basque population grew from 2,089,950 to 2,180,449. A limitation of OBI is that it does not contain data from private practice records. Even though access to the health system is nearly universal for all residents, 20% of the population has double or complementary coverage.

The study population were all individuals who, as of 31 December 2018, were between 1 and 30 years old and were registered in the Basque Health Service. Among this population, we identified patients with a diagnosis of mental health problems considering all the episodes of primary, emergency, outpatient and in-hospital care. Following the advice of experts (psychiatrists, psychologists, epidemiologists and medical documentalists), diagnoses were aggregated into eight clusters: anxiety (anxiety + acute stress reactions + adaptation reactions), attention deficit hyperactivity disorder (ADHD), conduct disorders, depression (depression + bipolar disorder), substance use, psychosis and personality disorders, eating disorders, and self-harm. In the identification process, the ICD-9-Clinical Modification and ICD-10 provided the framework codes as shown in Table SM1.

The variables included in the study were: age, sex, income level based on drug co-payment (see below), vital status (alive/dead), date of birth and death (in such cases), diagnoses, date of first diagnosis, medication prescribed, and medication prescription date. Given the dynamic nature of the study, the population varies over time with individuals entering or leaving it with the occurrence of certain events [26] including migration and death.

Information on SES was obtained based on drug co-payment categories which are established according to income of the designated parent. This classification does not take into account the whole household income but has been broadly used as a surrogate of the SES [20]. The most disadvantaged SES level (low SES) included children, adolescents and young people from households whose head was exempt from co-payment. The most advantaged SES level (high SES) corresponded to cases in which the head of the household had an annual income higher than €18,000, with a third category for annual incomes lower than this amount (medium SES) [20].

### Statistical analysis

All episodes of care associated with a mental disorder diagnosis in the Basque Health Service were identified. Individuals with various diagnoses were included in each corresponding cluster. Age at first episode related to a given diagnosis of each individual was recorded as age of onset for that cluster. Subsequently, for all clusters of mental health disorders, incidence rates were obtained for each year of age, overall and disaggregated by gender and SES (low, medium and high). For each year of age, the numerator of the incidence rate was obtained by summing the cases of the 30 cohorts, whose age of onset of a given diagnostic cluster was that age, distributing all the cases across years 1–30. For each year and disorder cluster, we estimated the denominator as the number of individual years of exposure. For that, all the individuals from the cohorts of the corresponding age or more were included, and the cases diagnosed in the previous years were subtracted from each cohort [26]. In other words, the population used as the denominator of the incidence was all 30 cohorts for age 1 and only the population of the oldest cohort (30 years) for age 30. We calculated the cumulative incidence using the Aalen-Johansen estimator for competing risks (death and file closure due to individuals having moved away) [27] (plotCIF procedure from R/CRAN/Epi/ library).

In the initial step, we compared the prevalence of mental disorders across sociodemographic groups by analysing the differences in categorical variables using the chi-square test and means of normally distributed continuous variables using Student's *t* test.

To calculate incidence rate ratios (IRRs), we used Poisson regression models with a robust estimate of variance to assess the effects of gender, age, and SES on annual rates of mental disorders [28] with the logarithm of person-years at risk as an offset. The main assumption of the Poisson distribution is that the sample mean and variance are equal. If this assumption was not satisfied, the data being over-dispersed, we used a negative binomial distribution in generalised linear models instead of a Poisson distribution. We explored models with and without a gender-SES interaction. Statistical analyses were carried out with Stata (version 13) and R (version 3.6.1). All parameters were estimated together with their 95% confidence intervals.

## Results

The population analysed comprised 609,281 individuals aged between 1 and 30 years (Supplementary Table SM2) for whom 9,486,853 person-years of observation were available. Of them, 96,060 (15.8%) had a mental health disorder documented in their medical record. The lowest socioeconomic (SES) level represented 3.7% of the total population, and among this group, 20.7% of the individuals had a mental disorder diagnosis.

**Fig. 1:**
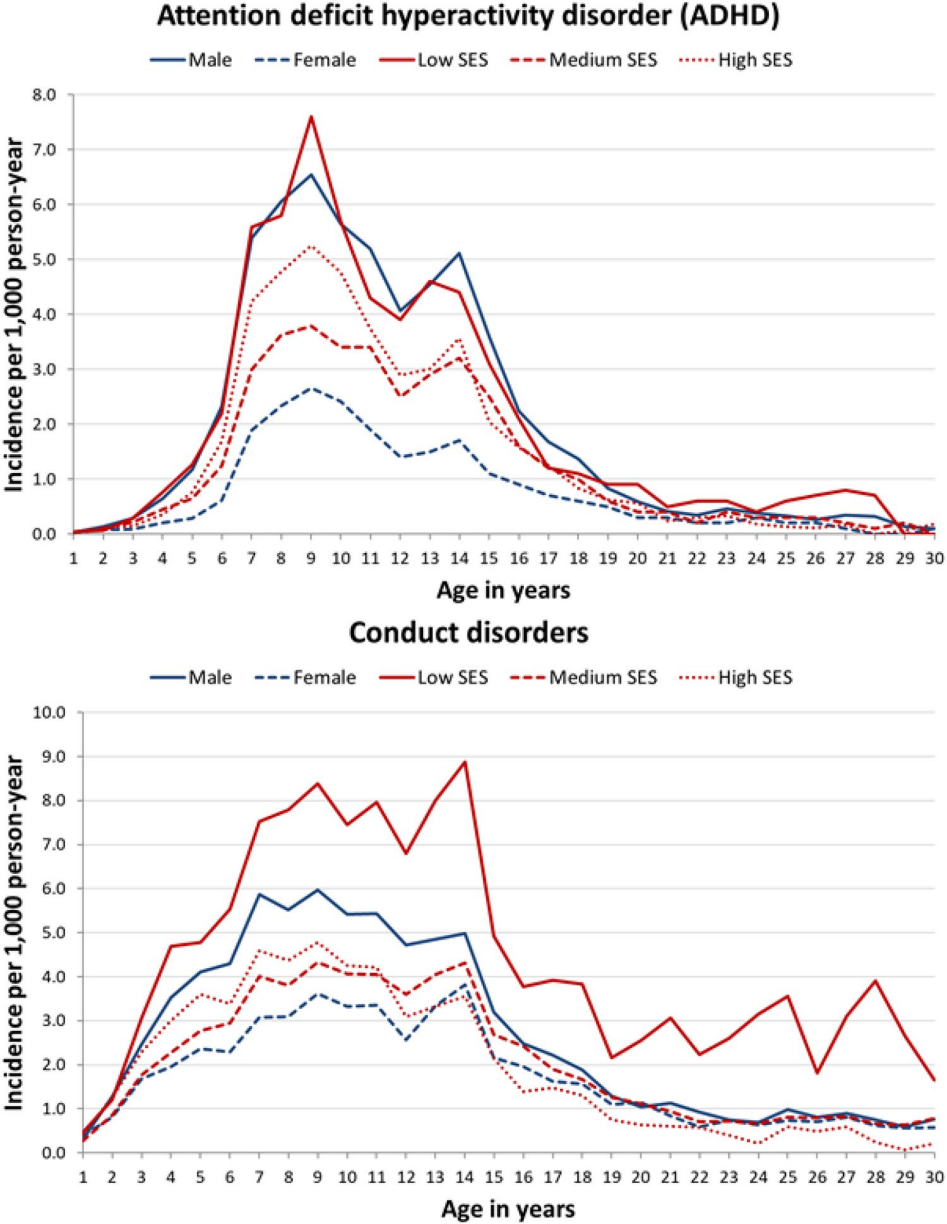
Incidence (cases/1000 person-year) of attention deficit hyperactivity disorder and conduct disorders disaggregated by gender and socioeconomic status. *SES* socioeconomic status

**Fig. 2:**
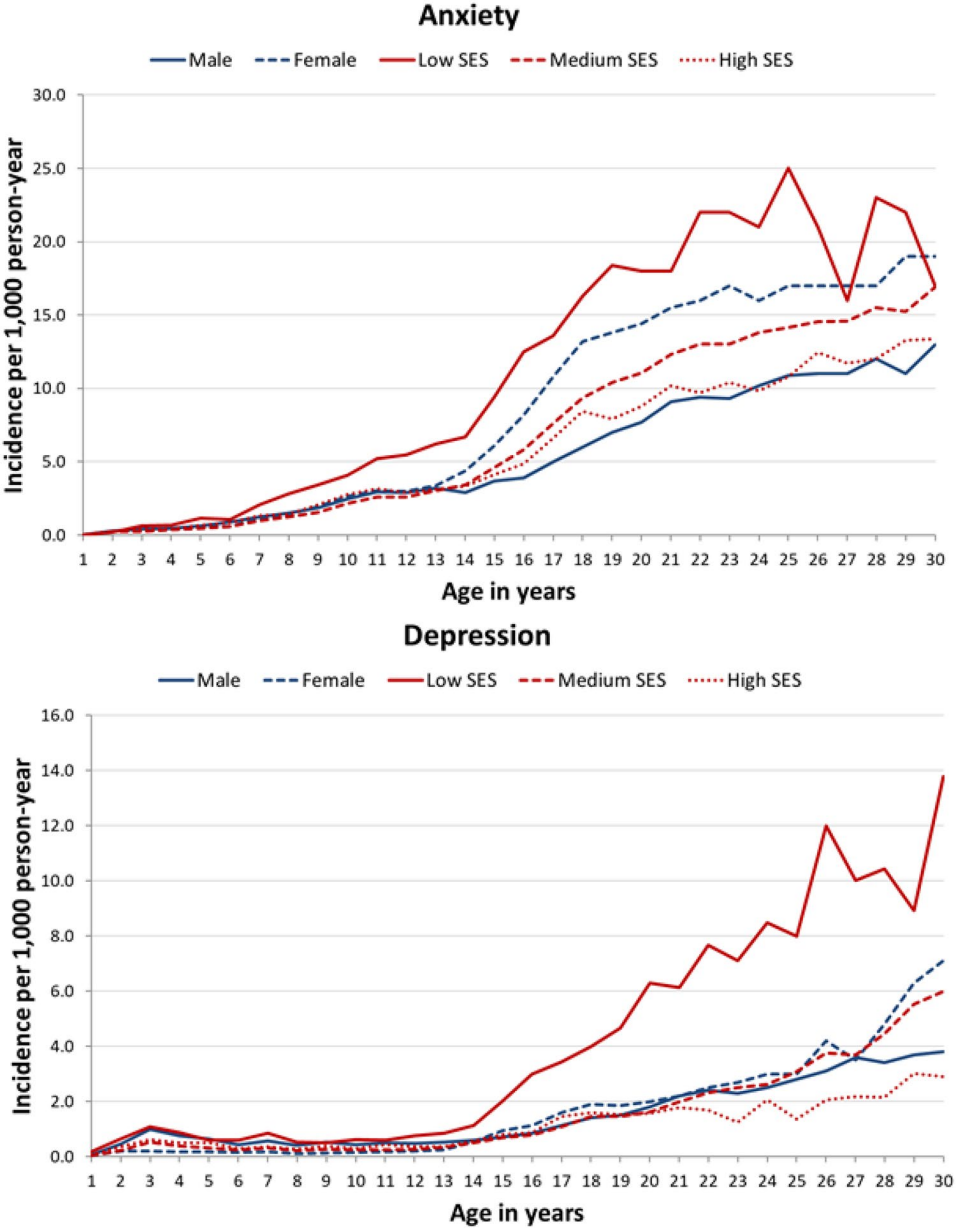
Incidence (cases/1000 person-year) of anxiety and depression disaggregated by gender and socioeconomic status. *SES* socioeconomic status

**Fig. 3:**
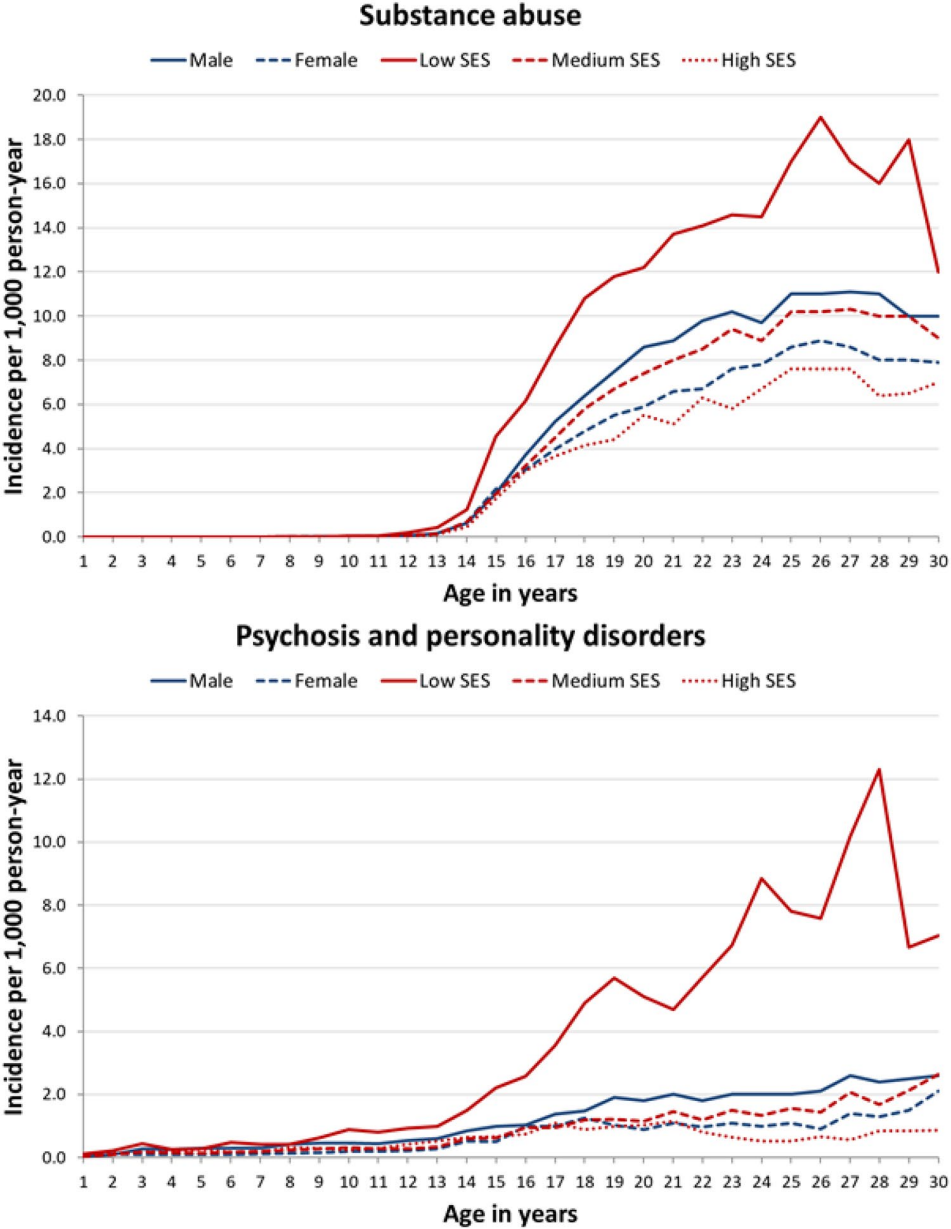
Incidence (cases/1000 person-year) of substance abuse and psychosis and personality disorders disaggregated by gender and socioeconomic status. *SES* socioeconomic status

Incidence rates are plotted by year of age and cluster of mental disorders measured in cases per 1000 person-years and disaggregated by gender and SES in Fig. 1 (ADHD and conduct disorders), Fig. 2 (anxiety and depression), Fig. 3 (psychosis and personality disorders, substance use) and Fig. SM1 (eating disorders, self-harm) in the Supplementary Material. Tables SM3–SM8 contain the same incidence rates (total, by gender and by SES). Anxiety was the cluster that presented the highest incidence, reaching 16 cases per 1000 person-years at 30 years of age, followed by substance use at 25 (9.9 per 1000 person-years) and depression at 26 (2.2 per 1000 person-years). In contrast, peaks in the incidence of ADHD and conduct disorders were observed between 7 and 14 years of age, with figures of around 4 cases per 1000 person-years.

Table 1 lists the cumulative incidence (%) in the eight clusters of mental disorders at 12, 18, 24 and 30 years of age, disaggregated by gender and SES. To facilitate interpretation of these results, we plot these same data at each year of age in Figs. SM2–SM9, in the Supplementary Material. At 18 years of age, this indicator gives a figure of 15.50% for any type of disorder, 3.87% for ADHD, 5.61% for conduct disorders, 5.07% for anxiety, 0.87% for depression, 1.66% for substance use, 0.62% for psychosis and personality disorders, 0.93% for eating disorders and 0.10% for self-harm. We also compared these results with data from a study with a similar design conducted in another European country, namely, the cumulative incidence at 18 years in Denmark stratified by gender [17] (Figs. SM10-SM11).

**Table 1:** Cumulative incidence (%) by type of mental disorder at 12, 18, 24 and 30 years of age, disaggregated by gender (women and men) and socioeconomic status (low, medium and high)

	Any disorder (%)	ADHD (%)	Conduct disorders (%)	Anxiety (%)	Depression (%)	Substance use (%)	Psychosis and personality disorders (%)	Eating disorders (%)	Self-harm (%)
Total									
12 years	8.25	2.60	3.90	1.54	0.41	0.01	0.21	0.36	0.01
18 years	15.50	3.87	5.61	5.07	0.87	1.66	0.62	0.93	0.10
24 years	25.68	4.11	6.15	12.30	1.79	6.40	1.31	1.30	0.21
30 years	36.57	4.21	6.59	21.10	2.89	12.10	2.02	1.51	0.35
Female									
12 years	6.03	1.39	2.85	1.52	0.19	0.01	0.14	0.37	0.01
18 years	13.84	2.04	4.30	6.13	0.67	1.49	0.52	1.35	0.15
24 years	25.45	2.22	4.80	15.40	1.57	5.50	0.99	2.00	0.27
30 years	37.60	2.28	5.20	26.00	2.73	10.50	1.47	2.36	0.44
Male									
12 years	10.36	3.75	4.89	1.57	0.62	0.01	0.28	0.35	0.01
18 years	17.07	5.61	6.85	4.04	1.06	1.82	0.72	0.53	0.05
24 years	25.88	5.91	7.44	9.31	2.00	7.29	1.63	0.63	0.15
30 years	35.56	6.06	7.91	16.20	3.04	13.70	2.55	0.68	0.27
Low SES									
12 years	12.38	2.84	6.90	2.05	0.96	0.07	0.50	0.58	0.02
18 years	25.29	3.87	10.82	9.21	2.44	4.47	2.25	1.37	0.25
24 years	43.06	4.26	12.83	23.52	5.90	15.00	5.88	1.80	0.88
30 years	57.99	4.55	14.54	37.60	8.76	25.70	8.82	2.22	1.91
Medium SES									
12 years	7.62	2.36	3.66	1.43	0.36	0.01	0.19	0.32	0.01
18 years	14.97	3.63	5.45	4.97	0.80	1.68	0.60	0.86	0.09
24 years	25.48	3.87	6.00	12.38	1.72	6.60	1.29	1.25	0.20
30 years	36.76	3.98	6.47	21.42	2.90	12.60	2.06	1.45	0.36
High SES									
12 years	8.81	2.94	3.98	1.68	0.44	0.00	0.21	0.40	0.01
18 years	15.35	4.22	5.34	4.82	0.85	1.34	0.51	1.00	0.10
24 years	23.58	4.45	5.68	10.70	1.48	4.83	0.87	1.32	0.14
30 years	32.95	4.52	5.90	18.03	2.13	9.12	1.10	1.52	0.16

*ADHD* attention deficit hyperactivity disorder, *SES *socioeconomic status

The results of the multivariable Poisson models analysing incidence rates and corresponding 95% confidence intervals are presented in Table 2 with a gender-SES interaction and Table SM10 without any interaction. Low SES was statistically significantly associated with a higher incidence of mental disorders for all clusters except ADHD, with an IRR of 1.84 for any disease, and an IRR of 2.04 for conduct disorders, 2.04 for anxiety, 3.54 for depression, 3.43 for substance use, 5.92 for psychosis and personality disorders, 1.33 for eating disorders and 5.46 for self-harm. Statistically significant differences in incidence were observed in males for any disorder (IRR = 1.14) but with differences according to the type of disorder, the risk being higher for ADHD (IRR = 2.74), conduct disorders (IRR = 1.62), substance use (IRR = 1.37), psychosis and personality disorders (IRR = 1.75) and depression (IRR = 1.46), and lower for anxiety (IRR = 0.63), eating disorders (IRR = 0.41) and self-harm (IRR = 0.65). When rates were compared by SES category (Table SM10), a marked difference was observed between the low SES and the high and medium SES groups, the IRR for all disorders being higher in the low SES group (1.84). Differences displayed by gender-SES interaction (Table 2) were noteworthy, low SES males, compared with high SES females, having much higher risks for mental disorders, with IRRs as high as 10.20 for psychosis and personality disorders, 7.22 for depression, 5.26 for substance use, 5.86 for self-harm, 2.72 for ADHD, 3.45 for conduct disorders and 1.45 for anxiety. The risk in the low SES group was only lower for the eating disorders cluster, with an IRR of 0.92 but without statistical significance.

**Table 2:** Incidence rate ratios of mental disorder as a function of age, gender and socioeconomic status (Poisson regressions of incidence rates with gender # SES interaction)

	Any disorder	ADHD	Conduct disorders	Anxiety	Depression	Substance use	Psychosis and personality	Eating disorders	Self-harm
Age IRR	1.09	1.00	0.99	1.16	1.10	1.23	1.12	1.05	1.16
CI (p)	1.09–1.09 (< 0.001)	1.00–1.01 (< 0.001)	0.99–0.99 (< 0.001)	1.16–1.16 (< 0.001)	1.09–1.10 (< 0.001)	1.22–1.23 (< 0.001)	1.11–1.12 (< 0.001)	1.04–1.05 (< 0.001)	1.15–1.17 (< 0.001)
Female # high SES IRR	Reference	Reference	Reference	Reference	Reference	Reference	Reference	Reference	Reference
Female # medium SES IRR	1.06	0.75	0.91	1.14	1.29	1.46	1.18	0.95	1.31
CI (p)	1.04–1.08 (< 0.001)	0.70–0.79 (< 0.001)	0.88–0.95 (< 0.001)	1.10–1.17 (< 0.001)	1.18–1.42 (< 0.001)	1.37–1.54 (< 0.001)	1.05–1.33 (0.006)	0.88–1.02 (0.148)	1.03–1.65 (0.025)
Female) # SES (low) IRR	1.72	0.81	1.76	1.96	2.70	2.82	4.22	1.11	3.50
CI (p)	1.64–1.79 (< 0.001)	0.69–0.96 (0.017)	1.62–1.92 (< 0.001)	1.84–2.08 (< 0.001)	2.29–3.19 (< 0.001)	2.56–3.12 (< 0.001)	3.53–5.05 (< 0.001)	0.92–1.34 (0.265)	2.41–5.08 (< 0.001)
Male # high SES IRR	1.22	2.57	1.54	0.65	1.69	1.28	1.40	0.48	0.60
CI (p)	1.19–1.25 (< 0.001)	2.43–2.71 (< 0.001)	1.48–1.60 (< 0.001)	0.63–0.68 (< 0.001)	1.53–1.87 (< 0.001)	1.20–1.37 (< 0.001)	1.23–1.60 (< 0.001)	0.43–0.53 (< 0.001)	0.44–0.83 (0.002)
Male # medium SES IRR	1.15	2.12	1.50	0.70	1.65	1.98	2.10	0.34	0.74
CI (p)	1.13–1.18 (< 0.001)	2.01–2.23 (< 0.001)	1.44–1.56 (< 0.001)	0.67–0.72 (< 0.001)	1.51–1.81 (< 0.001)	1.87–2.09 (< 0.001)	1.87–2.35 (< 0.001)	0.31–0.37 (< 0.001)	0.57–0.95 (0.020)
Male # low SES IRR	2.44	2.72	3.45	1.45	7.22	5.26	10.20	0.92	5.86
CI (p)	2.34–2.55 (< 0.001)	2.45–3.03 (< 0.001)	3.21–3.69 (< 0.001)	1.34–1.56 (< 0.001)	6.34–8.22 (< 0.001)	4.79–5.77 (< 0.001)	8.78–11.85 (< 0.001)	0.74–1.14 (0.449)	4.14–8.30 (< 0.001)

*SES* socioeconomic status, *IRR* incidence rate ratio, *CI* 95% confidence intervals, *ADHD* attention deficit hyperactivity disorder

## Discussion

The main contribution of this study is the estimation of the incidence of mental disorders, grouped in diagnosis clusters, in children, adolescents, and young adults disaggregated by gender and SES level in a registry that covers the entire population of the Basque Country. Moreover, we have identified gender inequalities and a marked social gradient in the frequency of mental disorders in the first three decades of life. Rates in all mental disorder clusters followed parallel courses over time, but the risks remained two- to three-fold higher in the lowest SES than in the highest SES groups. Although the incidence of mental disorders in young people by gender has been measured previously in Danish registries, Refs. [16, 17] to our knowledge, this is the first time that it has also been disaggregated by SES in a population database. The age span analysed, up to the age of 30, made it possible to identify disorders that were treated in the first decade (ADHD and conduct disorders), in the second and third (anxiety, depression and eating disorders) and mostly in the third (psychosis and personality disorder, substance use and self-harm). Our findings of differences in the rate, onset age and course of mental disorders by age, gender and SES help visualise their burden and suggest that addressing them is a public health priority [10–12, 23]. This is consistent with studies reporting the negative impact of the coronavirus disease 2019 (COVID-19) pandemic on child and adolescent mental health. There is a great need for interventions to promote mental health among children and adolescents, as well as parenting support programs [29].

Our results are consistent with those found in Denmark [17]. A study by Steinhausen et al. based on a nationwide registry in Denmark found a somewhat lower cumulative incidence for any mental disorder at 18 years of age (11.02%) [16]; however, another Danish study using the same source (electronic health records), by Dalsgaard et al. [17], obtained results similar to ours: cumulative incidence rates for any mental disorder at age 18 of 14.63% in females and 15.51% in males in Denmark compared to rates of 13.84% and 17.07% respectively in the Basque Country. Moreover, compared with this second study [17], remarkable similarities were found for most of the disorder clusters (Figs. SM10–SM11): ADHD (2.04% and 5.61% in the Basque Country versus 2.35% and 5.13% in Denmark), anxiety (6.13% and 4.04% versus 7.85% and 4.58%), depression (0.67% and 1.06% versus 2.54% and 1.01%), substance use (1.49% and 1.82% versus 1.53% and 1.63%) and eating disorders (1.35% and 0.53% versus 1.80% and 0.28%). On the other hand, less consistency was observed for conduct disorders (4.30% and 6.85% in the Basque Country versus 1.87% and 3.08% in Denmark) and psychosis and personality disorders (0.52% and 0.72% versus 1.81% and 0.78%), though this is likely attributable to the way in which diagnoses were grouped. Specifically, to compare with our results, we summed the rates in the two diagnostic groups from Dalsgaard et al. and hence, comorbidity between psychoses and personality disorders may partially explain the higher figures in Demark. In our classification, patients only counted once notwithstanding that they might have both diagnoses. Indeed, analysing by clusters implied some lack of grouping accuracy but this was required to be exhaustive in the classification. The fact that this means the inclusion of some diagnoses in the different clusters was forced partially limits the comparability with other registries. Nonetheless, the consistency with the aforementioned real-world data Danish findings supports the view that our results are valid [17]. Similar notable consistency was also found in other studies measuring the incidence of dementia in real-world data registries among Catalan, Basque and other European populations [30–33].

Although the incidence over time varies among the clusters, the disaggregation by SES and gender showed profiles that followed parallel or proportional courses. Further, while the absolute risk of mental disorders differed across the groups by SES and gender, the age of onset of the eight clusters was similar when they were disaggregated by gender and SES. The incidence rates in the low SES groups showed peaks and valleys which are attributable to the smaller sample size, this group representing 3.7% of the whole population. In contrast, for the groups with medium and high SES, the distribution of the incidence rates was smoother throughout the years of follow-up, these groups having more person-years of observation. Our results confirmed the three patterns of onset of mental disorders described in the literature depending on whether their first diagnoses are recorded in the first decade, in the second and third, or mainly in the third [9, 16, 17]. Clarifying whether inequalities by SES imply different ages for the onset of mental disorders is especially relevant in prevention since it has been shown that adolescents with low SES are less likely to use mental health services [34].

Despite the different methodological approaches used to report the results, in terms of lifetime risk and age-of-onset percentiles, our patterns in mental disorders are reasonably consistent with those described in survey-based studies [9]. The age of onset was earlier for each impulse-control disorder (age 7–15 years) than for any substance (age 19–23 years) or mood (age 25–32 years) disorders. The onset age range was narrower for impulse-control (1–6 years) and substance use (6–12 years) disorders than for any depression (25–26 years) [8].

Similar to previous reports, in our study, the onset of ADHD and conduct disorders associated with challenging behaviours and impulse control was observed in the first decade of life [9, 17, 35]. As in the study by Dalsgaard et al., a peak in incidence peak occurred before the age of 10 years for both clusters (ADHD and conduct disorders). The disorders in these clusters are also more common among males and the low SES group, the IRR of low SES males being twice that of females in the same SES group and that of individuals with medium-to-high SES.

Internalising disorders such as anxiety and depression shared a pattern of onset in both adolescents and young adults (second and third decades). Notably, individuals with low SES had a two- to three-fold higher risk of these disorders than those with medium-to-high SES, the positive significant interaction indicated that this risk was somewhat lower in females with low SES. Anxiety had an onset before adolescence, but the incidence peaked in young adults, increasing with age up to 30 years. This pattern is similar to that observed in Denmark, where there was also an increasing trend in incidence during adolescence [17]. The distribution of the onset of depression overlapped with that of the incidence of anxiety, as in the World Mental Health studies [8, 9]. Nonetheless, anxiety began to appear early in adolescence while depression began a little later, the incidence then increasing with age until the age of 30. Along with substance use, depression constitutes the cluster for which the incidence increases most with age. These results are consistent with those in the literature that indicate a low incidence of depression until adolescence followed by a linear increase until middle age [9].

Disorders prompting contact with the health system in the third decade of life such as psychosis and personality disorders and substance use disorder had a higher incidence in males. Further, although the incidence of psychosis only grew slightly with age in the population as a whole, it soared from the end of adolescence in men with low SES. In addition, substance use had a late onset during adolescence but its incidence grew in young adults, reaching a peak at 20–25 years of age, as described in the literature [14]. Inequalities in these two clusters were especially striking, males with low SES having 5- to 7-fold higher risks than females with medium-to-high SES, evidencing that the excess risk associated with SES was greater than that associated with gender.

The main benefit of disaggregated data on incidence is to facilitate the development of effective public health programs for the prevention of mental disorders. This implies addressing risk factors taking into account their multi-level nature due to the correlation between family, educational and community environments [12, 23]. Given the evidence of inequalities by gender and SES, two alternative types of intervention have been proposed [12]. On the one hand, the programs aimed at at-risk groups and, on the other, a population approach addressing all adolescents as the target population by seeking to strengthen their resilience [12]. The former could be more effective but with the penalty of stigmatising the target adolescents. Moreover, given the social gradient, implementing multi-component interventions for all adolescents and in their school and family environment would also reduce inequalities because the most deprived adolescents and youth are exposed to higher hazards of mental disorders [36].

Before the availability of registries based on electronic health records, the incidence of mental disorders was measured by repeated retrospective surveys, in which the diagnoses are reported by patients and which are hindered by response call bias [16, 17]. The strengths of our study approach relate to it helping to overcome these weaknesses by counting the ICD codes documented in electronic health records. First, the sample studied being a nationwide population, we are measuring the actual incidence. Second, it is possible to disaggregate results by social determinants. Third, the age range studied spanned the most common ages of onset of the mental disorders of interest.

On the other hand, our study has certain limitations. First, the assignment of co-payment in Spain only takes into account one of the parents. Strictly speaking, there is no information on the whole SES level of the household as we lack in the data base the category of annual income of the other parent. Moreover, the definition used for the category of low SES was quite restrictive as it corresponded to only 3.7% of the population. On the other hand, it showed notable sensitivity in identifying at-risk individuals. Second, the assessment of the age of onset of mental disorders relies on diagnoses being documented in health records. As Kessler et al. pointed out, the time between the onset of symptoms and the first contact with the health system can be long, especially for mild disorders [9]. It is estimated that only 40% of people with symptoms of mental illness seek treatment in the same year, based on samples from the entire general population [37]. Nonetheless, the time elapsed until help is first sought is inversely related to the age of onset [37], and this would support the use of the age at first documentation of a diagnosis as a proxy for incidence. Another limitation is the bias associated with the lack of validation of the diagnostic codes recorded in different healthcare settings. For diseases such as cancer, pathological findings can be used to validate registries’ records but that is not an option for mental health conditions. As in other studies using real-world data [16, 17, 20], our data are taken from the electronic health record, containing records from hospital admissions, outpatient clinics, emergency departments and primary care consultations. In addition, we also assigned depression and psychosis diagnoses to individuals with a chronic prescription of antidepressants and antipsychotics respectively, which could overestimate their incidence. The use of these heterogeneous sources means that we are putting together incident cases registered in psychiatric clinics with cases of low severity such as those only recorded in primary care. Nonetheless, each case represents a person who contacted the health service looking for help for some type of mental disorder. This heterogeneity underlines the need to assess the validity of real-world data registries to measure the epidemiology of mental disorders. In this context, the good fit found with Danish incidence strengthens the idea that real-world data is a valid source of information for mental health epidemiology.

Notwithstanding these limitations, our study underlines, on the one hand, the great weight of mental disorders due to their early onset in childhood, adolescence and youth and, on the other, the large inequalities in their incidence by gender and SES, especially in the case of depression and psychosis in low SES males. Taken together, these findings highlight the need to develop early policies for the prevention of mental disorders, seeking to minimise the associated suffering and improve the equity of health interventions.

## Supplementary Information

Below is the link to the electronic supplementary material.

Supplementary file1 (PDF 1452 KB)

## Data Availability

Data were provided by the Basque Health Service. Our data sharing agreement clearly stipulates that they cannot be shared with any third party.
