# Supplementary material for: Incidence of mental disorders in the general population aged 1–30 years disaggregated by gender and socioeconomic status
Source: Soc Psychiatry Psychiatr Epidemiol. 2023 Jan 24;58(6):961–71. doi: 10.1007/s00127-023-02425-z (PMC9872752; doi:10.1007/s00127-023-02425-z)

**Incidence of mental disorders in the general population aged 1 to 30 years disaggregated by gender and socioeconomic status. Supplementary material.**

Table SM 1 - ICD codes used for each category of mental disorder.

| | ICD-9 | ICD-10 |
|---|---|---|
| **Attention deficit hyperactivity disorder (ADHD)** | 314.xx | F90.xx |
| **Conduct disorders** | 312.0x-312.2x, 312.4x-312.9x, 313.xx | F91.xx-F99.xx |
| **Anxiety** | 300.xx, 308.xx-309.xx | F40.xx-F49.xx |
| **Depression** | 296.xx-299.xx, 300.4, 311 | F30.xx-F39.xx |
| **Substance abuse** | 303.xx-305.xx | F10.xx-F19.xx |
| **Psychosis and personality disorders** | 291.xx-292.xx, 295.xx<br>301.xx-302.xx, 312.3x | F20.xx-F29.xx<br>F60.xx-F69.xx |
| **Eating disorders** | 307.1x, 307.5x | F50.xx |
| **Self-harm** | V62.84, E95x.xx | R45.851, T14.91, X71.xx-X83.xx |

Table SM 2 - Characteristics of the Basque population aged between 1 and 30 years at December 31, 2018.

| | | Study population | | Mental disorder diagnosed | | | | p-value[a] |
|---|---|---|---|---|---|---|---|---|
| | | | | No | | Yes | | |
| | | **N** | **%** | **N** | **%** | **N** | **%** | |
| Patients | Total | 609,381 | | 513,321 | 84.2% | 96,060 | 15.8% | |
| Age | Mean | 15.57 | | 14.70 | | 20.23 | | 0.000 |
| | 0-12 | 246,275 | 40.4% | 231,131 | 93.9% | 15,144 | 6.1% | 0.000 |
| | 13-18 | 123,109 | 20.2% | 100,726 | 81.8% | 22,383 | 18.2% | |
| | 19-24 | 114,710 | 18.8% | 88,093 | 76.8% | 26,617 | 23.2% | |
| | 25-30 | 125,287 | 20.6% | 93,371 | 74.5% | 31,916 | 25.5% | |
| Gender | Female | 296,556 | 48.7% | 252,151 | 85.0% | 44,405 | 15.0% | 0.000 |
| | Male | 312,825 | 51.3% | 261,170 | 83.5% | 51,655 | 16.5% | |
| Socioeconomic status | Low | 22,285 | 3.7% | 17,681 | 79.3% | 4,604 | 20.7% | 0.000 |
| | Medium | 329,993 | 54.2% | 268,219 | 81.3% | 61,774 | 18.7% | |
| | High | 257,103 | 42.2% | 227,421 | 88.5% | 29,682 | 11.5% | |

[a] *Chi square test was used for categorical variables and Student's t test for continuous variables.*

Table SM3 - Distribution of prevalence of mental disorders by copayment categories in the Basque population 1-30 years.

| Socioeconomic status | Code | Income | N | % | Mental disorders (n) | Mental disorders (%) |
|---|---|---|---|---|---|---|
| Low | TSI 001 | no income | 22,285 | 3.7% | 4,655 | 20.9% |
| Medium | TSI 002 retired | <18000 | 17,858 | 2.9% | 4,322 | 24.2% |
| | TSI 003 worker | <18000 | 312,135 | 51.2% | 57,829 | 18.5% |
| High | TSI 002 retired | >18000 | 7,273 | 1.2% | 1,494 | 20.5% |
| | TSI 004 worker | >18000 | 240,996 | 39.5% | 27,606 | 11.5% |
| | TSI 005 worker | >100000 | 6,952 | 1.1% | 535 | 7.7% |
| | TSI 006 worker | muface | 1,882 | 0.3% | 230 | 12.2% |
| Total | | | 609,381 | 100.0% | 96,671 | 15.9% |

Table SM 4 - Total incidence rates (cases/1000 person-years) by type of mental disorder per year.

| Age | Any disorder | ADHD | Conduct disorders | Anxiety | Depression | Substance use disorders | Psychosis and personality disorders | Eating disorders | Self-harm |
|---|---|---|---|---|---|---|---|---|---|
| **1** | 0.53 | 0.03 | 0.34 | 0.01 | 0.05 | 0.00 | 0.04 | 0.05 | 0.00 |
| **2** | 2.41 | 0.11 | 1.05 | 0.26 | 0.32 | 0.00 | 0.11 | 0.59 | 0.01 |
| **3** | 3.75 | 0.19 | 2.09 | 0.36 | 0.61 | 0.00 | 0.19 | 0.47 | 0.00 |
| **4** | 4.45 | 0.44 | 2.76 | 0.41 | 0.47 | 0.00 | 0.17 | 0.38 | 0.01 |
| **5** | 5.27 | 0.74 | 3.26 | 0.60 | 0.42 | 0.00 | 0.19 | 0.32 | 0.01 |
| **6** | 5.94 | 1.49 | 3.32 | 0.74 | 0.30 | 0.00 | 0.16 | 0.28 | 0.01 |
| **7** | 9.57 | 3.69 | 4.51 | 1.21 | 0.38 | 0.00 | 0.18 | 0.27 | 0.00 |
| **8** | 9.85 | 4.21 | 4.34 | 1.46 | 0.26 | 0.00 | 0.20 | 0.23 | 0.01 |
| **9** | 11.09 | 4.70 | 4.82 | 1.90 | 0.33 | 0.02 | 0.18 | 0.25 | 0.00 |
| **10** | 10.48 | 4.10 | 4.40 | 2.55 | 0.30 | 0.01 | 0.22 | 0.23 | 0.00 |
| **11** | 10.34 | 3.50 | 4.42 | 2.99 | 0.32 | 0.03 | 0.22 | 0.23 | 0.00 |
| **12** | 8.86 | 2.80 | 3.67 | 2.90 | 0.32 | 0.05 | 0.22 | 0.29 | 0.03 |
| **13** | 9.71 | 3.00 | 4.11 | 3.40 | 0.34 | 0.15 | 0.29 | 0.42 | 0.05 |
| **14** | 11.29 | 3.50 | 4.42 | 3.60 | 0.49 | 0.66 | 0.48 | 0.77 | 0.14 |
| **15** | 11.04 | 2.40 | 2.69 | 4.80 | 0.68 | 2.06 | 0.54 | 1.02 | 0.13 |
| **16** | 11.70 | 1.60 | 2.23 | 6.10 | 0.79 | 3.38 | 0.78 | 1.18 | 0.18 |
| **17** | 13.63 | 1.20 | 1.93 | 7.80 | 1.04 | 4.54 | 0.93 | 1.31 | 0.18 |
| **18** | 15.08 | 1.00 | 1.73 | 9.60 | 1.27 | 5.70 | 1.10 | 1.02 | 0.23 |
| **19** | 15.47 | 0.70 | 1.20 | 10.30 | 1.25 | 6.50 | 1.13 | 0.79 | 0.15 |
| **20** | 15.98 | 0.40 | 1.09 | 11.00 | 1.36 | 7.30 | 1.12 | 0.70 | 0.19 |
| **21** | 17.21 | 0.30 | 0.99 | 12.20 | 1.50 | 7.70 | 1.27 | 0.60 | 0.14 |
| **22** | 17.71 | 0.30 | 0.76 | 12.80 | 1.70 | 8.30 | 1.09 | 0.70 | 0.22 |
| **23** | 17.60 | 0.40 | 0.74 | 13.00 | 1.60 | 8.90 | 1.20 | 0.50 | 0.20 |
| **24** | 17.85 | 0.30 | 0.67 | 13.00 | 1.80 | 8.70 | 1.10 | 0.40 | 0.18 |
| **25** | 18.57 | 0.30 | 0.86 | 14.00 | 1.80 | 9.90 | 1.20 | 0.50 | 0.21 |
| **26** | 18.63 | 0.20 | 0.76 | 14.00 | 2.20 | 9.90 | 1.00 | 0.40 | 0.23 |
| **27** | 17.96 | 0.20 | 0.85 | 14.00 | 1.90 | 9.90 | 1.30 | 0.20 | 0.21 |
| **28** | 17.80 | 0.20 | 0.68 | 15.00 | 2.00 | 9.30 | 1.20 | 0.30 | 0.27 |
| **29** | 18.02 | 0.10 | 0.57 | 15.00 | 1.80 | 9.00 | 1.30 | 0.30 | 0.41 |
| **30** | 17.95 | 0.00 | 0.67 | 16.00 | 1.30 | 9.00 | 1.10 | 0.40 | 0.14 |

*ADHD: Attention deficit hyperactivity disorder.*

Table SM 5 - Incidence rates (cases/1000 person-years) by type of mental disorder per year for males.

| Age | Any disorder | ADHD | Conduct disorders | Anxiety | Depression | Substance use disorders | Psychosis and personality disorders | Eating disorders | Self-harm |
|---|---|---|---|---|---|---|---|---|---|
| **1** | 0.49 | 0.03 | 0.30 | 0.02 | 0.06 | 0.00 | 0.04 | 0.03 | 0.00 |
| **2** | 2.78 | 0.14 | 1.28 | 0.29 | 0.45 | 0.00 | 0.10 | 0.57 | 0.01 |
| **3** | 4.74 | 0.29 | 2.47 | 0.41 | 0.99 | 0.00 | 0.27 | 0.56 | 0.00 |
| **4** | 5.78 | 0.65 | 3.52 | 0.46 | 0.76 | 0.00 | 0.24 | 0.41 | 0.01 |
| **5** | 6.75 | 1.18 | 4.11 | 0.66 | 0.64 | 0.00 | 0.28 | 0.29 | 0.00 |
| **6** | 7.89 | 2.32 | 4.29 | 0.88 | 0.44 | 0.00 | 0.24 | 0.27 | 0.01 |
| **7** | 12.70 | 5.39 | 5.86 | 1.27 | 0.58 | 0.00 | 0.24 | 0.29 | 0.00 |
| **8** | 12.70 | 6.06 | 5.52 | 1.50 | 0.40 | 0.00 | 0.29 | 0.20 | 0.01 |
| **9** | 13.83 | 6.55 | 5.97 | 1.88 | 0.53 | 0.02 | 0.24 | 0.26 | 0.00 |
| **10** | 12.63 | 5.65 | 5.42 | 2.46 | 0.44 | 0.01 | 0.25 | 0.21 | 0.00 |
| **11** | 12.59 | 5.20 | 5.43 | 2.97 | 0.46 | 0.02 | 0.28 | 0.20 | 0.01 |
| **12** | 10.67 | 4.06 | 4.72 | 2.90 | 0.46 | 0.04 | 0.29 | 0.18 | 0.02 |
| **13** | 11.31 | 4.54 | 4.85 | 3.20 | 0.48 | 0.17 | 0.36 | 0.23 | 0.03 |
| **14** | 11.91 | 5.12 | 4.98 | 2.90 | 0.52 | 0.64 | 0.52 | 0.29 | 0.09 |
| **15** | 10.60 | 3.59 | 3.20 | 3.70 | 0.61 | 1.97 | 0.63 | 0.27 | 0.07 |
| **16** | 10.00 | 2.24 | 2.47 | 3.90 | 0.69 | 3.72 | 0.73 | 0.37 | 0.08 |
| **17** | 11.25 | 1.68 | 2.22 | 5.00 | 0.88 | 5.21 | 1.01 | 0.39 | 0.09 |
| **18** | 12.04 | 1.37 | 1.89 | 6.00 | 1.22 | 6.40 | 1.15 | 0.27 | 0.08 |
| **19** | 12.84 | 0.84 | 1.29 | 7.00 | 1.20 | 7.50 | 1.48 | 0.19 | 0.12 |
| **20** | 13.73 | 0.59 | 1.05 | 7.70 | 1.40 | 8.60 | 1.57 | 0.14 | 0.16 |
| **21** | 15.07 | 0.41 | 1.13 | 9.10 | 1.80 | 8.90 | 1.60 | 0.20 | 0.11 |
| **22** | 15.50 | 0.34 | 0.93 | 9.40 | 1.70 | 9.80 | 1.40 | 0.19 | 0.22 |
| **23** | 15.37 | 0.47 | 0.75 | 9.30 | 1.60 | 10.20 | 1.60 | 0.19 | 0.19 |
| **24** | 15.65 | 0.37 | 0.69 | 10.20 | 1.70 | 9.70 | 1.50 | 0.11 | 0.14 |
| **25** | 16.73 | 0.34 | 0.98 | 10.90 | 1.80 | 11.00 | 1.40 | 0.18 | 0.18 |
| **26** | 16.54 | 0.26 | 0.81 | 11.00 | 2.30 | 11.00 | 1.50 | 0.15 | 0.27 |
| **27** | 16.01 | 0.34 | 0.90 | 11.00 | 2.20 | 11.10 | 1.80 | 0.04 | 0.17 |
| **28** | 16.49 | 0.32 | 0.75 | 12.00 | 1.70 | 11.00 | 1.60 | 0.05 | 0.24 |
| **29** | 15.16 | 0.14 | 0.59 | 11.00 | 1.50 | 10.00 | 1.70 | 0.04 | 0.30 |
| **30** | 15.87 | 0.10 | 0.76 | 13.00 | 0.90 | 10.00 | 1.20 | 0.07 | 0.12 |

*ADHD: Attention deficit hyperactivity disorder.*

Table SM 6 - Incidence rates (cases/1000 person-years) by type of mental disorder per year for females.

| Age | Any disorder | ADHD | Conduct disorders | Anxiety | Depression | Substance use disorders | Psychosis and personality disorders | Eating disorders | Self-harm |
|---|---|---|---|---|---|---|---|---|---|
| **1** | 0.56 | 0.03 | 0.38 | 0.01 | 0.04 | 0.00 | 0.03 | 0.06 | 0.00 |
| **2** | 2.02 | 0.08 | 0.81 | 0.23 | 0.19 | 0.00 | 0.12 | 0.61 | 0.01 |
| **3** | 2.70 | 0.09 | 1.68 | 0.31 | 0.21 | 0.00 | 0.10 | 0.39 | 0.00 |
| **4** | 3.04 | 0.21 | 1.96 | 0.37 | 0.17 | 0.00 | 0.09 | 0.34 | 0.01 |
| **5** | 3.71 | 0.29 | 2.36 | 0.54 | 0.18 | 0.00 | 0.09 | 0.35 | 0.01 |
| **6** | 3.89 | 0.62 | 2.30 | 0.59 | 0.15 | 0.00 | 0.08 | 0.30 | 0.00 |
| **7** | 6.26 | 1.89 | 3.08 | 1.15 | 0.17 | 0.00 | 0.11 | 0.25 | 0.00 |
| **8** | 6.86 | 2.33 | 3.09 | 1.41 | 0.10 | 0.00 | 0.11 | 0.25 | 0.00 |
| **9** | 8.19 | 2.66 | 3.61 | 1.93 | 0.12 | 0.01 | 0.13 | 0.25 | 0.00 |
| **10** | 8.20 | 2.41 | 3.33 | 2.63 | 0.15 | 0.00 | 0.18 | 0.25 | 0.00 |
| **11** | 7.97 | 1.90 | 3.35 | 3.04 | 0.18 | 0.03 | 0.15 | 0.27 | 0.00 |
| **12** | 6.94 | 1.40 | 2.56 | 3.00 | 0.18 | 0.06 | 0.16 | 0.41 | 0.03 |
| **13** | 8.01 | 1.50 | 3.34 | 3.40 | 0.20 | 0.14 | 0.21 | 0.61 | 0.07 |
| **14** | 10.63 | 1.70 | 3.83 | 4.40 | 0.44 | 0.68 | 0.43 | 1.26 | 0.20 |
| **15** | 11.51 | 1.10 | 2.15 | 6.10 | 0.76 | 2.16 | 0.45 | 1.82 | 0.19 |
| **16** | 13.49 | 0.90 | 1.96 | 8.20 | 0.89 | 3.03 | 0.84 | 2.01 | 0.29 |
| **17** | 16.14 | 0.70 | 1.63 | 10.80 | 1.21 | 3.97 | 0.84 | 2.27 | 0.27 |
| **18** | 18.30 | 0.60 | 1.56 | 13.20 | 1.32 | 4.80 | 1.05 | 1.80 | 0.39 |
| **19** | 18.24 | 0.50 | 1.10 | 13.80 | 1.31 | 5.50 | 0.77 | 1.50 | 0.19 |
| **20** | 18.35 | 0.30 | 1.14 | 14.40 | 1.24 | 5.90 | 0.69 | 1.20 | 0.23 |
| **21** | 19.45 | 0.30 | 0.84 | 15.50 | 1.38 | 6.60 | 0.92 | 1.10 | 0.15 |
| **22** | 20.04 | 0.20 | 0.60 | 16.00 | 1.50 | 6.70 | 0.80 | 1.10 | 0.21 |
| **23** | 19.93 | 0.20 | 0.73 | 17.00 | 1.60 | 7.60 | 0.84 | 0.90 | 0.22 |
| **24** | 20.15 | 0.30 | 0.64 | 16.00 | 2.00 | 7.80 | 0.73 | 0.70 | 0.21 |
| **25** | 20.49 | 0.20 | 0.74 | 17.00 | 1.80 | 8.60 | 0.87 | 0.80 | 0.26 |
| **26** | 20.81 | 0.20 | 0.70 | 17.00 | 2.10 | 8.90 | 0.40 | 0.50 | 0.18 |
| **27** | 19.97 | 0.10 | 0.81 | 17.00 | 1.70 | 8.60 | 0.80 | 0.50 | 0.25 |
| **28** | 19.15 | 0.00 | 0.62 | 17.00 | 2.20 | 8.00 | 0.90 | 0.50 | 0.29 |
| **29** | 20.94 | 0.00 | 0.56 | 19.00 | 2.10 | 8.00 | 0.90 | 0.60 | 0.53 |
| **30** | 20.05 | 0.10 | 0.57 | 19.00 | 1.70 | 7.90 | 0.90 | 0.70 | 0.16 |

*ADHD: Attention deficit hyperactivity disorder.*

Table SM 7 - Incidence rates (cases/1000 person-years) by type of mental disorder per year for low socioeconomic status.

| Age | Any disorder | ADHD | Conduct disorders | Anxiety | Depression | Substance use disorders | Psychosis and personality disorders | Eating disorders | Self-harm |
|---|---|---|---|---|---|---|---|---|---|
| **1** | 0.81 | 0.00 | 0.54 | 0.00 | 0.09 | 0.00 | 0.18 | 0.00 | 0.00 |
| **2** | 3.58 | 0.05 | 1.33 | 0.14 | 0.88 | 0.00 | 0.27 | 0.92 | 0.05 |
| **3** | 6.23 | 0.19 | 3.31 | 0.58 | 1.20 | 0.00 | 0.62 | 0.72 | 0.00 |
| **4** | 8.02 | 0.71 | 5.45 | 0.65 | 1.00 | 0.00 | 0.26 | 0.46 | 0.05 |
| **5** | 8.30 | 1.34 | 4.68 | 0.75 | 0.75 | 0.00 | 0.48 | 0.75 | 0.00 |
| **6** | 9.40 | 2.09 | 6.31 | 0.34 | 0.78 | 0.00 | 0.28 | 0.23 | 0.00 |
| **7** | 15.80 | 4.78 | 7.80 | 2.02 | 1.30 | 0.00 | 0.54 | 0.47 | 0.00 |
| **8** | 13.94 | 4.55 | 7.71 | 2.03 | 0.57 | 0.00 | 0.38 | 0.32 | 0.00 |
| **9** | 15.98 | 5.20 | 8.52 | 2.31 | 0.61 | 0.00 | 0.27 | 0.69 | 0.00 |
| **10** | 13.40 | 3.70 | 7.70 | 2.72 | 0.72 | 0.07 | 0.58 | 0.15 | 0.00 |
| **11** | 15.59 | 2.90 | 8.95 | 4.95 | 0.63 | 0.08 | 0.32 | 0.56 | 0.00 |
| **12** | 12.70 | 2.90 | 6.73 | 3.96 | 1.04 | 0.52 | 0.77 | 0.52 | 0.09 |
| **13** | 13.85 | 2.20 | 7.59 | 6.17 | 0.73 | 0.86 | 0.84 | 0.75 | 0.19 |
| **14** | 18.61 | 2.60 | 9.56 | 6.68 | 1.20 | 2.45 | 1.21 | 2.04 | 0.31 |
| **15** | 20.69 | 2.40 | 6.29 | 8.90 | 2.70 | 6.42 | 2.87 | 1.00 | 0.55 |
| **16** | 24.38 | 1.40 | 5.45 | 15.22 | 3.30 | 8.10 | 3.34 | 1.20 | 0.48 |
| **17** | 24.97 | 1.20 | 5.59 | 16.46 | 3.00 | 11.40 | 4.31 | 1.70 | 0.39 |
| **18** | 26.60 | 0.50 | 4.69 | 18.22 | 3.90 | 14.80 | 5.01 | 1.28 | 0.44 |
| **19** | 31.39 | 0.80 | 2.82 | 22.11 | 5.30 | 16.90 | 7.24 | 0.46 | 0.48 |
| **20** | 25.95 | 1.00 | 3.55 | 20.91 | 6.00 | 15.20 | 5.28 | 0.85 | 1.36 |
| **21** | 29.48 | 0.70 | 4.05 | 22.18 | 4.70 | 17.40 | 5.88 | 0.55 | 0.19 |
| **22** | 33.38 | 0.20 | 2.83 | 28.00 | 6.40 | 17.80 | 6.05 | 0.81 | 1.20 |
| **23** | 30.81 | 0.50 | 2.27 | 24.48 | 5.10 | 19.00 | 4.38 | 1.09 | 1.76 |
| **24** | 26.74 | 0.70 | 4.65 | 25.47 | 7.10 | 19.00 | 7.40 | 0.48 | 1.22 |
| **25** | 31.82 | 0.50 | 4.12 | 29.50 | 6.10 | 18.00 | 6.67 | 1.34 | 1.65 |
| **26** | 26.49 | 0.90 | 1.75 | 23.68 | 7.70 | 21.00 | 4.10 | 0.32 | 1.20 |
| **27** | 20.36 | 1.10 | 3.31 | 19.65 | 5.00 | 17.00 | 6.97 | 0.00 | 1.20 |
| **28** | 27.81 | 0.40 | 3.74 | 25.27 | 3.60 | 19.00 | 7.76 | 0.42 | 1.90 |
| **29** | 23.95 | 0.00 | 3.43 | 24.03 | 4.60 | 17.00 | 1.91 | 1.10 | 2.90 |
| **30** | 18.91 | 0.00 | 0.71 | 18.60 | 1.60 | 15.00 | 2.05 | 1.08 | 1.50 |

*ADHD: Attention deficit hyperactivity disorder.*

Table SM 8 - Incidence rates (cases/1000 person-years) by type of mental disorder per year for medium socioeconomic status.

| Age | Any disorder | ADHD | Conduct disorders | Anxiety | Depression | Substance use disorders | Psychosis and personality disorders | Eating disorders | Self-harm |
|---|---|---|---|---|---|---|---|---|---|
| **1** | 0.43 | 0.04 | 0.27 | 0.00 | 0.05 | 0.00 | 0.03 | 0.04 | 0.00 |
| **2** | 1.99 | 0.10 | 0.87 | 0.23 | 0.25 | 0.00 | 0.09 | 0.47 | 0.00 |
| **3** | 3.33 | 0.21 | 1.85 | 0.28 | 0.55 | 0.00 | 0.17 | 0.41 | 0.00 |
| **4** | 4.00 | 0.48 | 2.40 | 0.39 | 0.43 | 0.00 | 0.15 | 0.34 | 0.00 |
| **5** | 4.65 | 0.67 | 2.92 | 0.53 | 0.32 | 0.00 | 0.14 | 0.29 | 0.01 |
| **6** | 5.44 | 1.30 | 3.07 | 0.66 | 0.28 | 0.00 | 0.16 | 0.25 | 0.01 |
| **7** | 8.56 | 3.19 | 4.23 | 1.05 | 0.33 | 0.00 | 0.16 | 0.23 | 0.00 |
| **8** | 9.09 | 3.82 | 4.07 | 1.39 | 0.24 | 0.00 | 0.17 | 0.20 | 0.00 |
| **9** | 10.18 | 4.08 | 4.61 | 1.74 | 0.29 | 0.02 | 0.19 | 0.22 | 0.00 |
| **10** | 9.81 | 3.60 | 4.25 | 2.35 | 0.28 | 0.01 | 0.24 | 0.25 | 0.00 |
| **11** | 9.95 | 3.50 | 4.28 | 2.78 | 0.26 | 0.02 | 0.21 | 0.21 | 0.00 |
| **12** | 8.79 | 2.60 | 3.80 | 2.86 | 0.29 | 0.06 | 0.18 | 0.26 | 0.03 |
| **13** | 9.89 | 3.00 | 4.34 | 3.27 | 0.34 | 0.15 | 0.27 | 0.40 | 0.06 |
| **14** | 11.34 | 3.40 | 4.55 | 3.65 | 0.48 | 0.68 | 0.47 | 0.73 | 0.15 |
| **15** | 11.27 | 2.50 | 2.78 | 4.92 | 0.65 | 2.03 | 0.52 | 1.00 | 0.11 |
| **16** | 11.86 | 1.60 | 2.46 | 6.09 | 0.74 | 3.35 | 0.81 | 1.07 | 0.18 |
| **17** | 13.69 | 1.20 | 1.96 | 7.85 | 0.97 | 4.68 | 0.89 | 1.21 | 0.15 |
| **18** | 15.41 | 1.00 | 1.75 | 9.65 | 1.21 | 5.80 | 1.11 | 1.07 | 0.22 |
| **19** | 15.97 | 0.70 | 1.29 | 10.60 | 1.17 | 6.80 | 1.08 | 0.86 | 0.16 |
| **20** | 16.50 | 0.40 | 1.15 | 11.20 | 1.27 | 7.50 | 1.06 | 0.70 | 0.18 |
| **21** | 17.72 | 0.30 | 0.99 | 12.39 | 1.60 | 8.10 | 1.29 | 0.60 | 0.15 |
| **22** | 18.19 | 0.30 | 0.74 | 13.11 | 1.60 | 8.50 | 1.12 | 0.70 | 0.24 |
| **23** | 18.18 | 0.30 | 0.77 | 13.04 | 1.70 | 9.40 | 1.30 | 0.60 | 0.21 |
| **24** | 18.60 | 0.40 | 0.65 | 13.72 | 1.90 | 8.90 | 1.10 | 0.40 | 0.15 |
| **25** | 18.97 | 0.30 | 0.82 | 14.11 | 1.90 | 10.10 | 1.20 | 0.50 | 0.21 |
| **26** | 18.96 | 0.20 | 0.80 | 14.47 | 2.20 | 10.20 | 1.00 | 0.30 | 0.25 |
| **27** | 18.81 | 0.20 | 0.84 | 14.50 | 2.10 | 10.40 | 1.40 | 0.30 | 0.21 |
| **28** | 18.40 | 0.20 | 0.71 | 15.42 | 2.20 | 10.30 | 1.20 | 0.30 | 0.29 |
| **29** | 18.64 | 0.10 | 0.63 | 15.25 | 2.00 | 9.00 | 1.70 | 0.20 | 0.46 |
| **30** | 18.95 | 0.10 | 0.81 | 16.73 | 1.40 | 10.00 | 1.20 | 0.40 | 0.14 |

*ADHD: Attention deficit hyperactivity disorder.*

Table SM 9 - Incidence rates (cases/1000 person-years) by type of mental disorder per year for high socioeconomic status.

| Age | Any disorder | ADHD | Conduct disorders | Anxiety | Depression | Substance use disorders | Psychosis and personality disorders | Eating disorders | Self-harm |
|---|---|---|---|---|---|---|---|---|---|
| **1** | 0.62 | 0.02 | 0.42 | 0.04 | 0.05 | 0.00 | 0.03 | 0.05 | 0.01 |
| **2** | 2.85 | 0.11 | 1.25 | 0.31 | 0.37 | 0.00 | 0.13 | 0.72 | 0.01 |
| **3** | 4.08 | 0.16 | 2.29 | 0.45 | 0.63 | 0.00 | 0.17 | 0.55 | 0.01 |
| **4** | 4.74 | 0.36 | 3.01 | 0.43 | 0.49 | 0.00 | 0.19 | 0.42 | 0.01 |
| **5** | 5.84 | 0.79 | 3.60 | 0.68 | 0.50 | 0.00 | 0.23 | 0.32 | 0.00 |
| **6** | 6.35 | 1.69 | 3.42 | 0.88 | 0.29 | 0.00 | 0.15 | 0.34 | 0.01 |
| **7** | 10.47 | 4.31 | 4.62 | 1.38 | 0.37 | 0.00 | 0.17 | 0.31 | 0.00 |
| **8** | 10.62 | 4.85 | 4.45 | 1.50 | 0.27 | 0.00 | 0.24 | 0.24 | 0.02 |
| **9** | 12.04 | 5.40 | 4.84 | 2.13 | 0.36 | 0.01 | 0.16 | 0.27 | 0.00 |
| **10** | 11.29 | 4.80 | 4.37 | 2.83 | 0.30 | 0.00 | 0.16 | 0.20 | 0.00 |
| **11** | 10.55 | 3.90 | 4.27 | 3.23 | 0.38 | 0.03 | 0.22 | 0.24 | 0.01 |
| **12** | 8.67 | 3.00 | 3.22 | 2.95 | 0.35 | 0.00 | 0.25 | 0.34 | 0.02 |
| **13** | 9.07 | 3.10 | 3.44 | 3.17 | 0.30 | 0.11 | 0.27 | 0.42 | 0.02 |
| **14** | 10.62 | 3.70 | 3.74 | 3.42 | 0.43 | 0.48 | 0.44 | 0.72 | 0.12 |
| **15** | 9.76 | 2.10 | 2.19 | 4.27 | 0.59 | 1.79 | 0.39 | 1.10 | 0.12 |
| **16** | 10.24 | 1.60 | 1.43 | 5.10 | 0.66 | 3.02 | 0.49 | 1.38 | 0.16 |
| **17** | 12.49 | 1.30 | 1.52 | 6.83 | 1.03 | 3.78 | 0.70 | 1.55 | 0.24 |
| **18** | 13.17 | 1.00 | 1.37 | 8.61 | 1.16 | 4.20 | 0.70 | 0.82 | 0.26 |
| **19** | 12.42 | 0.60 | 0.77 | 8.34 | 1.09 | 4.60 | 0.67 | 0.80 | 0.08 |
| **20** | 13.37 | 0.60 | 0.65 | 9.23 | 0.88 | 5.80 | 0.83 | 0.60 | 0.13 |
| **21** | 14.16 | 0.20 | 0.64 | 10.50 | 1.30 | 5.20 | 0.69 | 0.60 | 0.04 |
| **22** | 14.22 | 0.40 | 0.61 | 9.87 | 1.00 | 6.50 | 0.63 | 0.50 | 0.02 |
| **23** | 13.99 | 0.30 | 0.44 | 10.76 | 0.70 | 6.00 | 0.44 | 0.40 | 0.00 |
| **24** | 14.17 | 0.20 | 0.21 | 10.09 | 1.30 | 6.80 | 0.30 | 0.30 | 0.13 |
| **25** | 15.66 | 0.20 | 0.59 | 10.82 | 0.90 | 7.60 | 0.34 | 0.40 | 0.07 |
| **26** | 16.72 | 0.10 | 0.48 | 12.34 | 1.30 | 7.70 | 0.35 | 0.40 | 0.04 |
| **27** | 15.23 | 0.10 | 0.58 | 11.59 | 1.30 | 7.70 | 0.38 | 0.20 | 0.08 |
| **28** | 15.02 | 0.00 | 0.24 | 11.91 | 1.00 | 6.50 | 0.38 | 0.30 | 0.00 |
| **29** | 15.72 | 0.10 | 0.07 | 13.24 | 1.00 | 6.50 | 0.50 | 0.40 | 0.00 |
| **30** | 15.34 | 0.20 | 0.29 | 13.46 | 1.00 | 6.90 | 0.40 | 0.30 | 0.00 |

*ADHD: Attention deficit hyperactivity disorder.*

Table SM10 - Incidence rate ratios of mental disorder as a function of age, gender and socioeconomic status (Poisson regressions of incidence rates without gender # SES interaction).

| | **Any disorder** | **ADHD** | **Conduct disorders** | **Anxiety** | **Depression** | **Substance abuse** | **Psychosis and personality disorders** | **Eating disorders** | **Self-harm** |
|---|---|---|---|---|---|---|---|---|---|
| Age IRR | 1.09 | 1.00 | 0.99 | 1.16 | 1.10 | 1.23 | 1.12 | 1.05 | 1.16 |
| CI (p) | 1.09-1.09(<0.001) | 1.00-1.01(<0.001) | 0.99-0.99(<0.001) | 1.16-1.16(<0.001) | 1.09-1.10(<0.001) | 1.22-1.23(<0.001) | 1.11-1.12(<0.001) | 1.04-1.05(<0.001) | 1.15-1.17(<0.001) |
| Female IRR | Reference | Reference | Reference | Reference | Reference | Reference | Reference | Reference | Reference |
| Male IRR | 1.14 | 2.74 | 1.62 | 0.63 | 1.46 | 1.37 | 1.75 | 0.41 | 0.65 |
| CI (p) | 1.13-1.16(<0.001) | 2.65-2.84(<0.001) | 1.58-1.66(<0.001) | 0.61-0.64(<0.001) | 1.39-1.54(<0.001) | 1.33-1.41(<0.001) | 1.65-1.86(<0.001) | 0.39-0.44(<0.001) | 0.56-0.76(<0.001) |
| SES high IRR | Reference | Reference | Reference | Reference | Reference | Reference | Reference | Reference | Reference |
| SES medium IRR | 1.00 | 0.81 | 0.95 | 1.11 | 1.09 | 1.51 | 1.38 | 0.87 | 1.28 |
| CI (p) | 0.98-1.01(0.521) | 0.78-0.83(<0.001) | 0.93-0.98(<0.001) | 1.08-1.14(<0.001) | 1.03-1.16(0,002) | 1.45-1.56(<0.001) | 1.28-1.48(<0.001) | 0.82-0.92(<0.001) | 1.07-1.54(0,007) |
| SES low IRR | 1.84 | 0.99 | 2.04 | 2.04 | 3.54 | 3.43 | 5.92 | 1.33 | 5.46 |
| CI (p) | 1.79-1.90(<0.001) | 0.91-1.08(0.774) | 1.94-2.15(<0.001) | 1.94-2.14(<0.001) | 3.22-3.90(<0.001) | 3.21-3.67(<0.001) | 5.31-6.60(<0.001) | 1.16-1.54(<0.001) | 4.20-7.09(<0.001) |

SES: socio economic status; IRR: incidence rate ratio; CI: 95% confidence intervals; ADHD: attention deficit hyperactivity disorder.

Figure SM 1 - Incidence (cases/1,000 person-year) of eating disorders and self-harm disaggregated by gender and socioeconomic status.

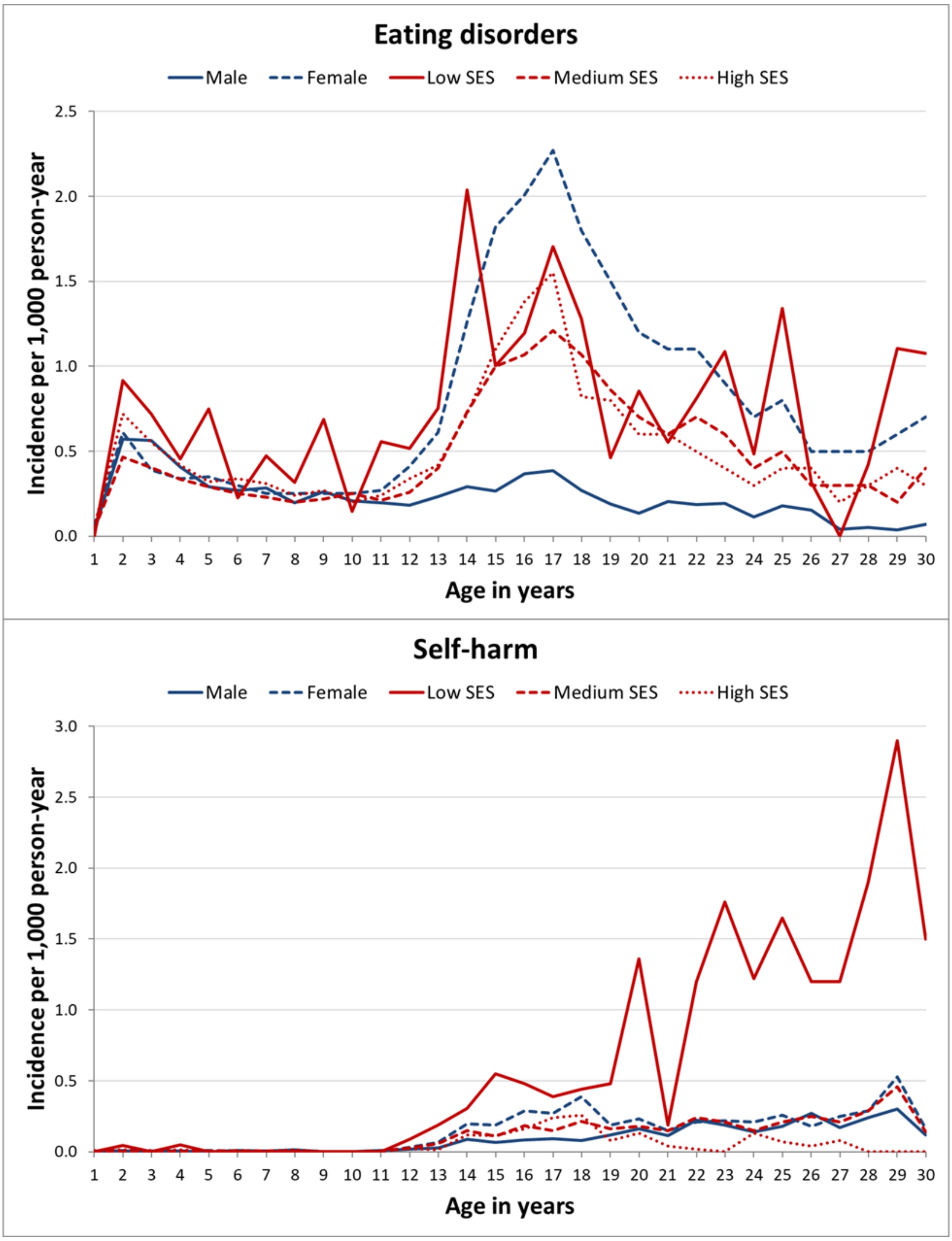

Figure SM2 - Cumulative incidence (%) of ADHD disaggregated by gender and socioeconomic status.

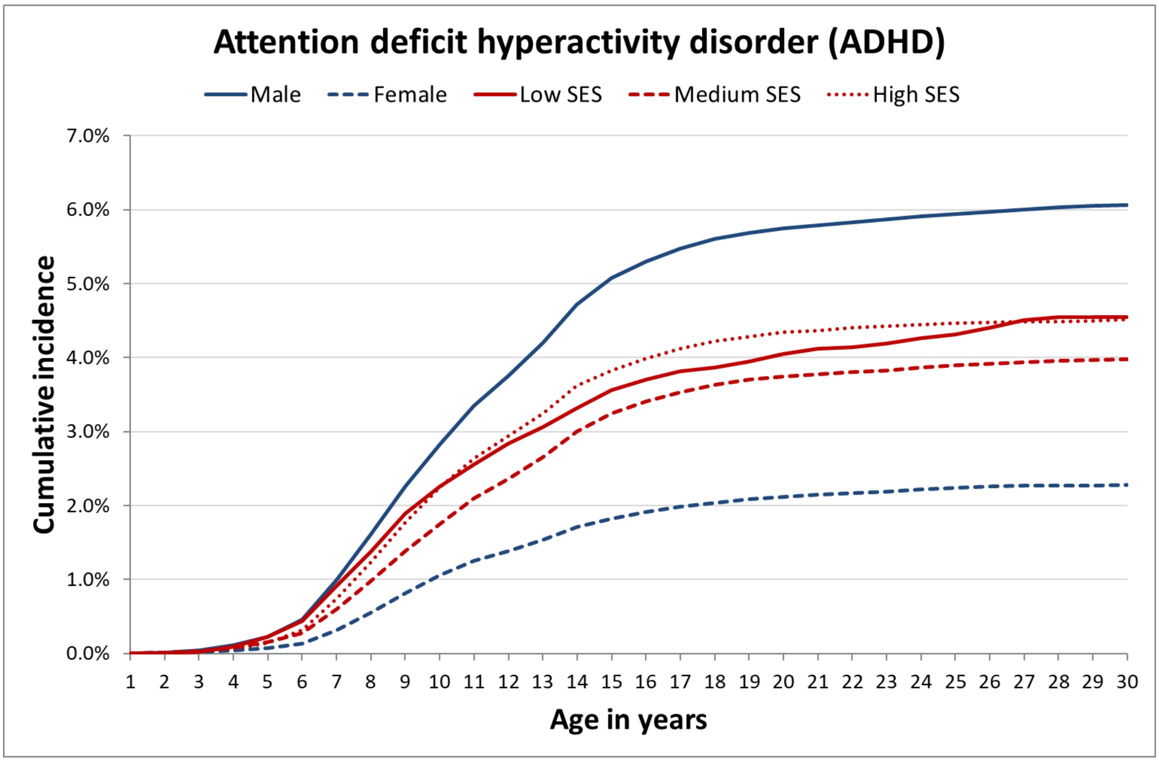

Figure SM3 - Cumulative incidence (%) of conduct disorders disaggregated by gender and socioeconomic status.

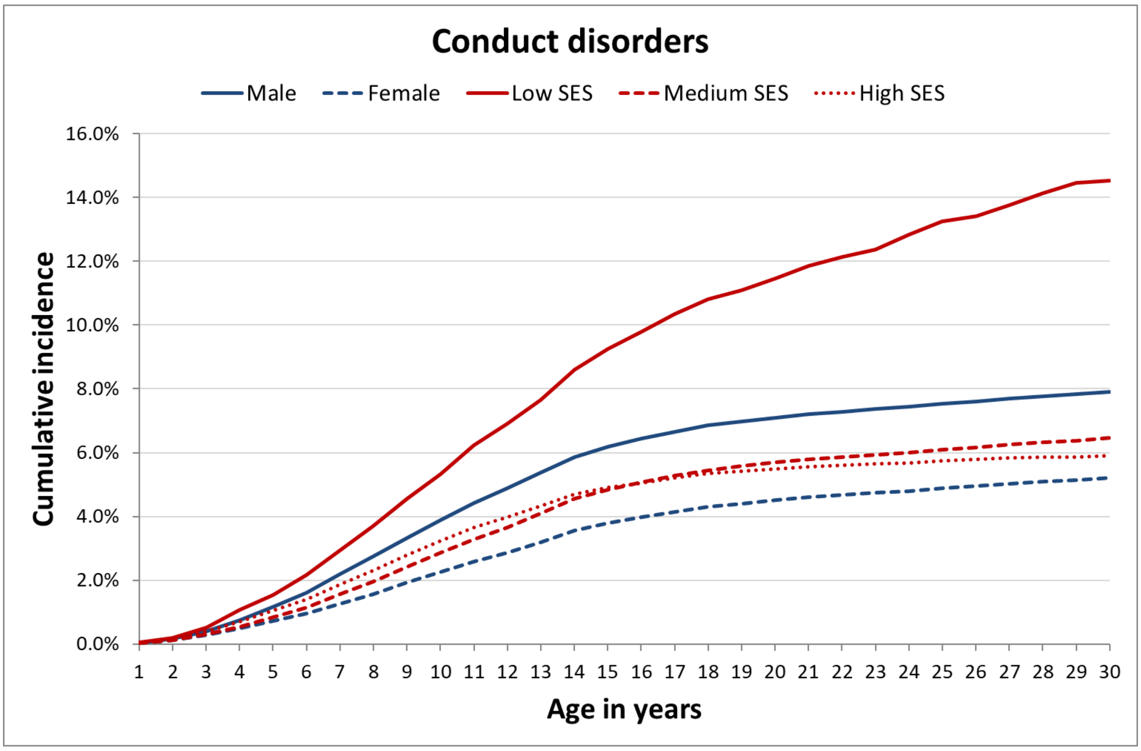

Figure SM4 - Cumulative incidence (%) of anxiety disaggregated by gender and socioeconomic status.

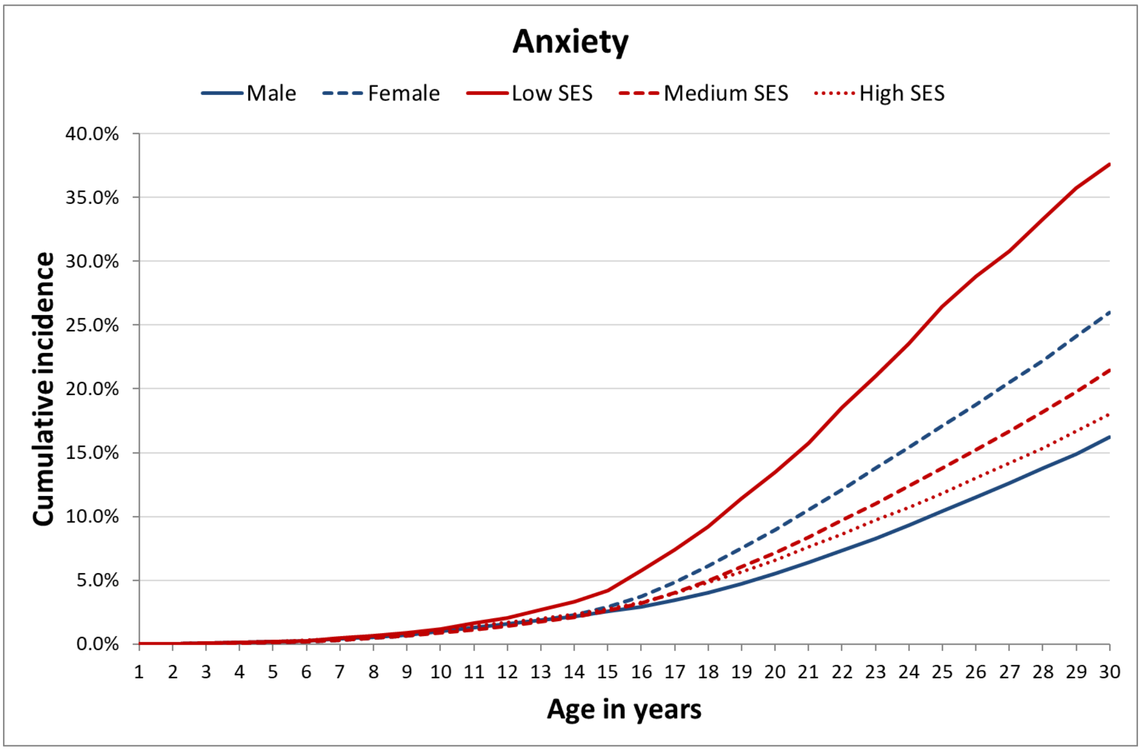

Figure SM5 - Cumulative incidence (%) of depression disaggregated by gender and socioeconomic status.

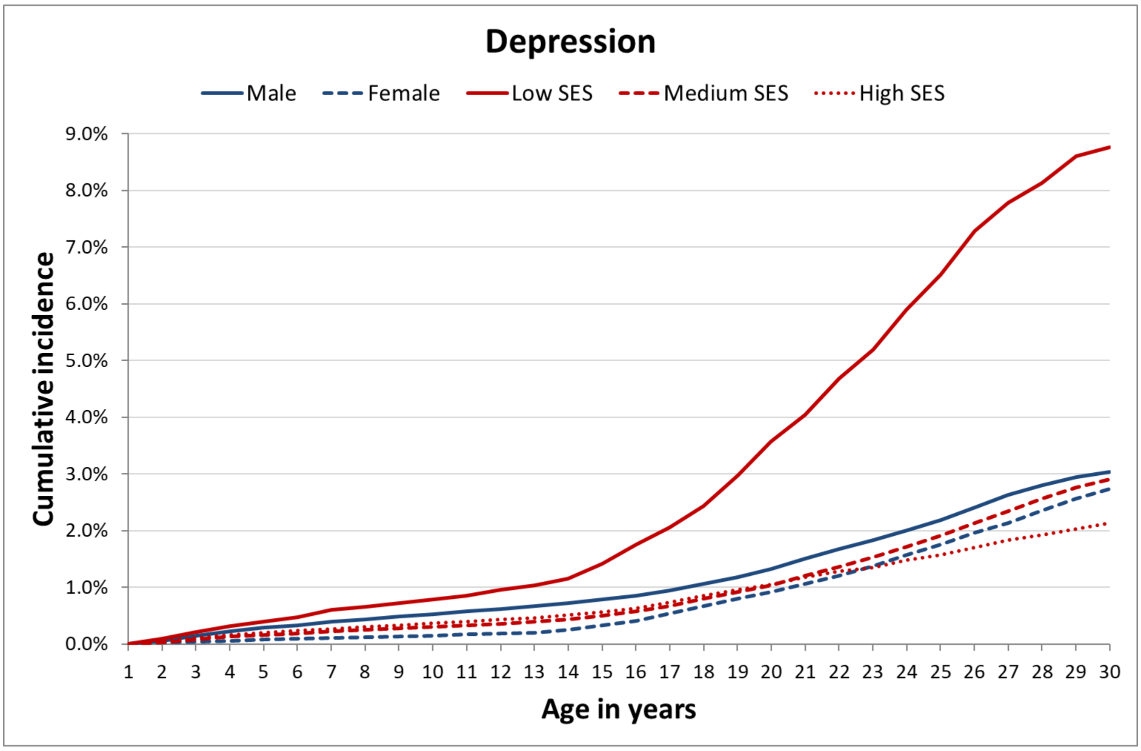

Figure SM6 - Cumulative incidence (%) of substance abuse disaggregated by gender and socioeconomic status.

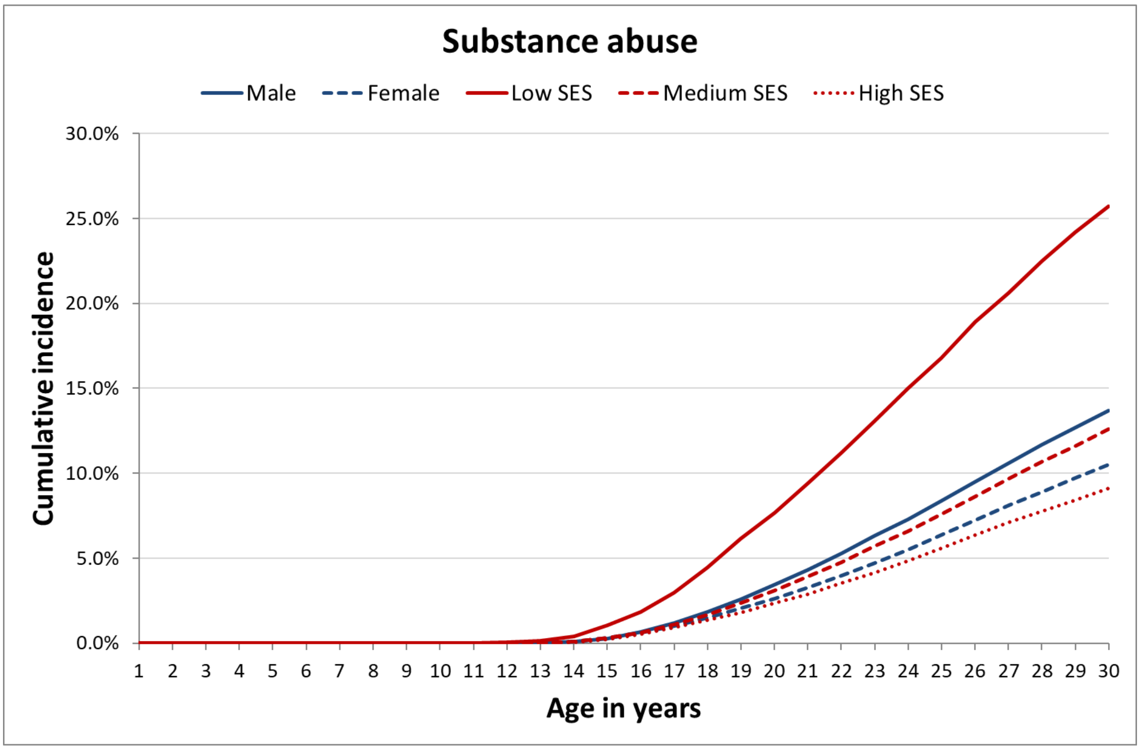

Figure SM7 - Cumulative incidence (%) of psychosis and personality disorders disaggregated by gender and socioeconomic status.

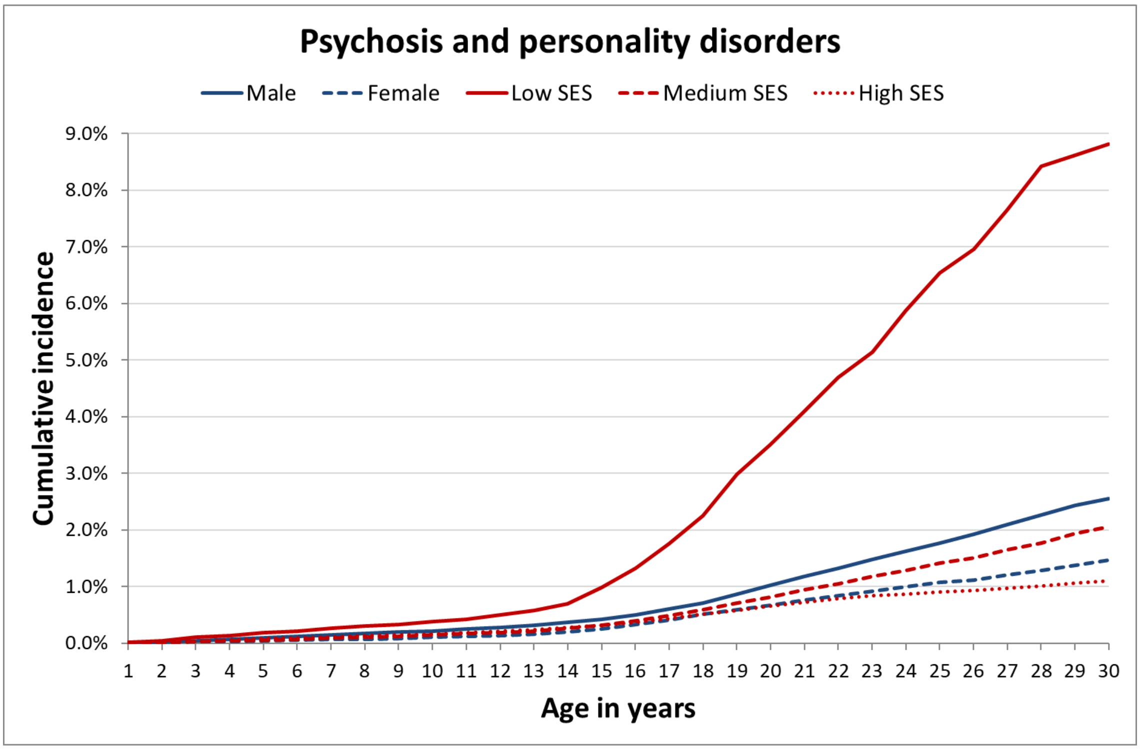

Figure SM8 - Cumulative incidence (%) of eating disorders disaggregated by gender and socioeconomic status.

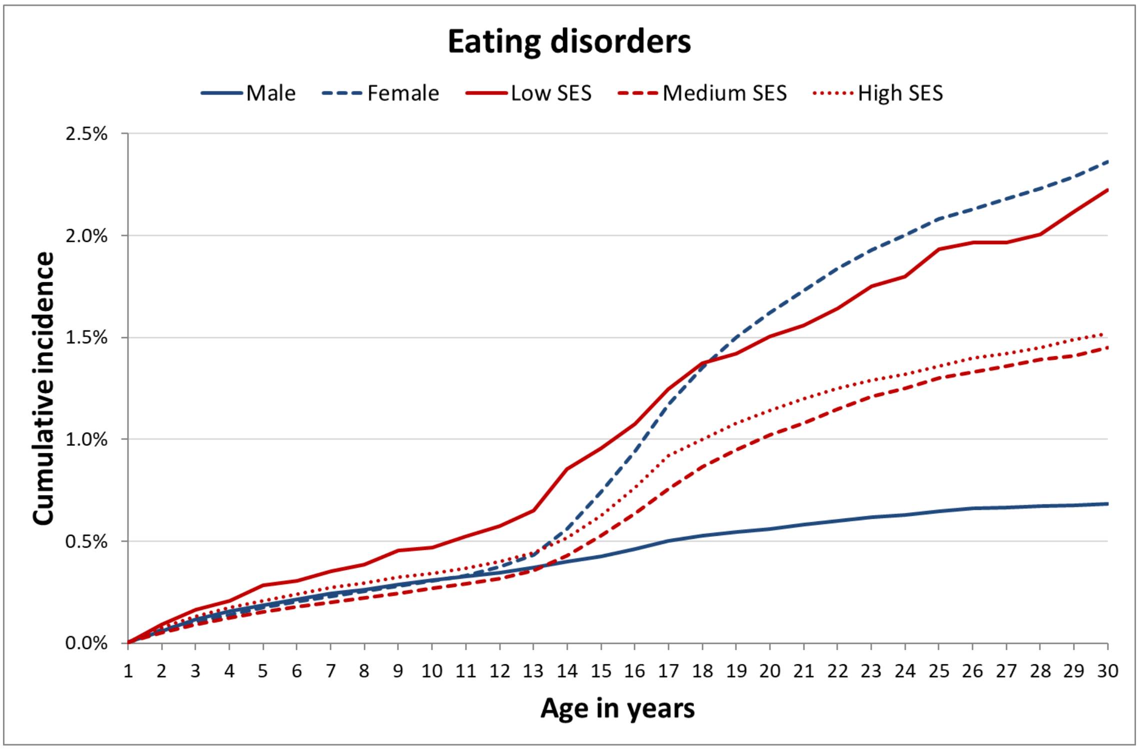

Figure SM9 - Cumulative incidence (%) of self-harm disaggregated by gender and socioeconomic status.

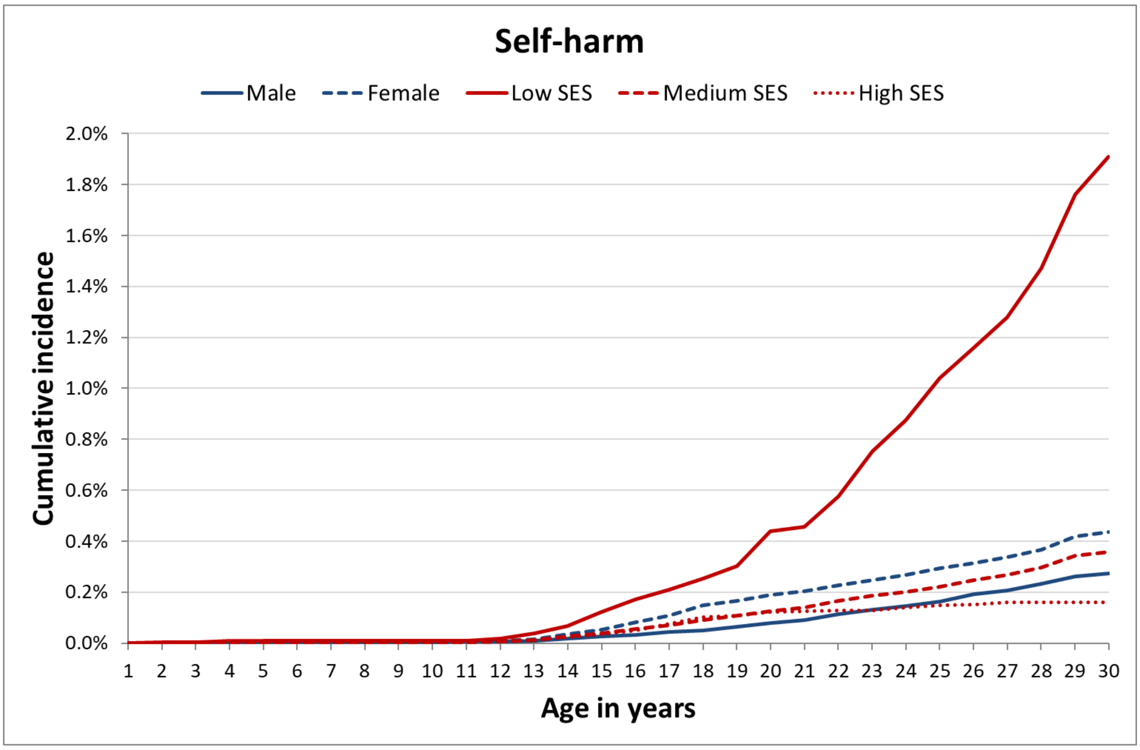

Figure SM10 - Comparison of the cumulative incidence (%) of mental disorders in Denmark and Basque country in females at the age of 18.

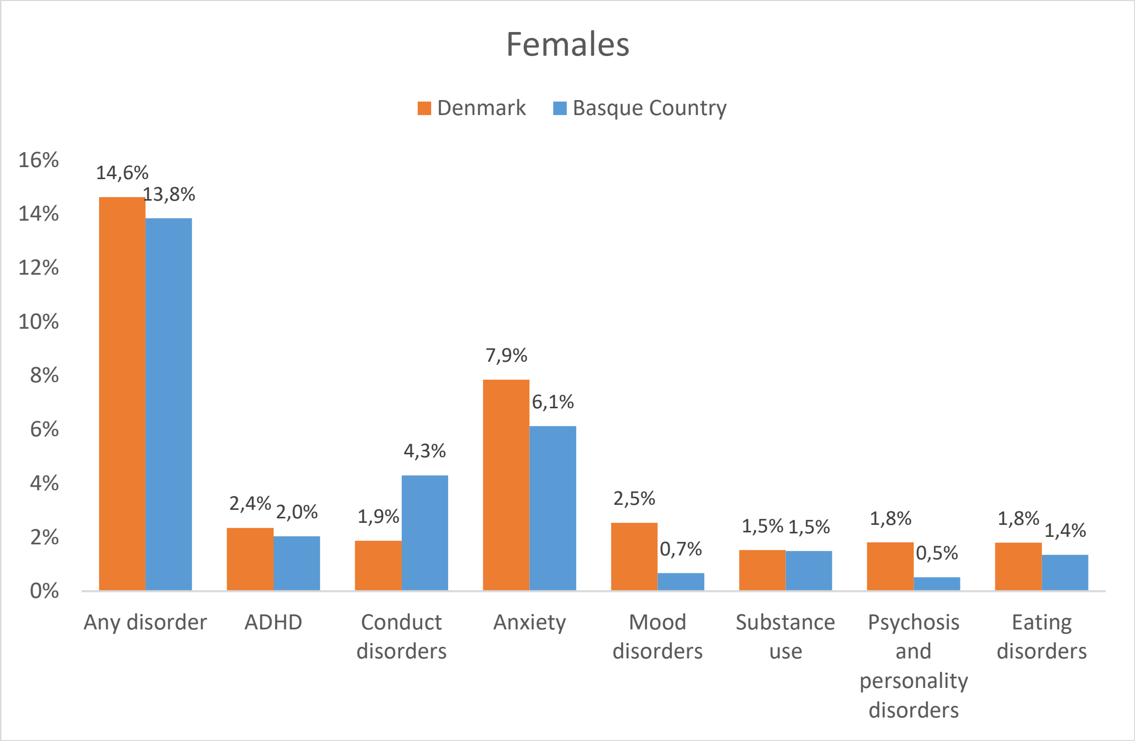

Figure SM11 - Comparison of the cumulative incidence (%) of mental disorders in Denmark and Basque country in males at the age of 18.

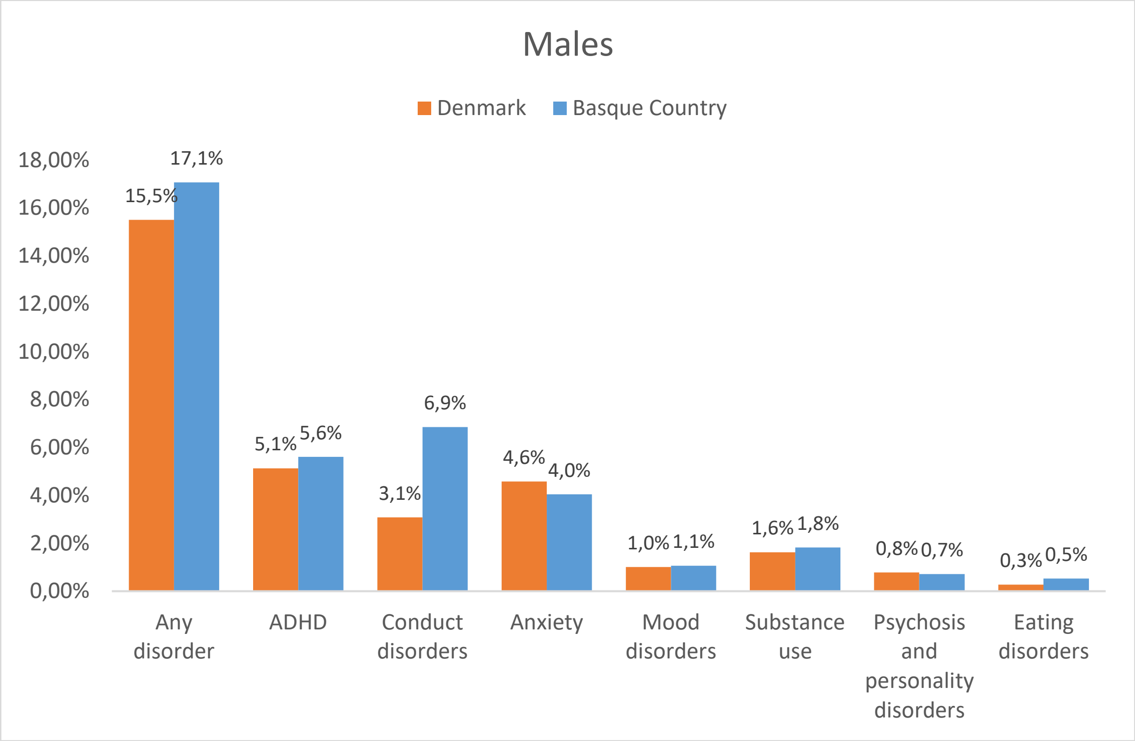

Supplement: Supplementary file 1 — Supplementary file1 (PDF 1452 KB) [file 127_2023_2425_MOESM1_ESM.pdf]
